# Sequence and Expression Analyses of Ethylene Response Factors Highly Expressed in Latex Cells from *Hevea brasiliensis*

**DOI:** 10.1371/journal.pone.0099367

**Published:** 2014-06-27

**Authors:** Piyanuch Piyatrakul, Meng Yang, Riza-Arief Putranto, Julien Pirrello, Florence Dessailly, Songnian Hu, Marilyne Summo, Kannikar Theeravatanasuk, Julie Leclercq, Kuswanhadi, Pascal Montoro

**Affiliations:** 1 Unité mixte de recherche Amélioration Génétique et Adaptation des Plantes méditerranéennes et tropicales, Centre International de Recherche Agronomique pour le Développement, Montpellier, France; 2 Department of Agriculture, Rubber Research Institute, Bangkok, Thailand; 3 Beijing Institute of Genomics, Chinese Academy of Science, Beijing, China; 4 Indonesian Biotechnology Research Institute for Estate Crops, Bogor, Indonesia; 5 Sembawa Research Center, Indonesian Rubber Research Institute, Palembang, Indonesia; ISA, Portugal

## Abstract

The AP2/ERF superfamily encodes transcription factors that play a key role in plant development and responses to abiotic and biotic stress. In *Hevea brasiliensis*, *ERF* genes have been identified by RNA sequencing. This study set out to validate the number of *HbERF* genes, and identify ERF genes involved in the regulation of latex cell metabolism. A comprehensive *Hevea* transcriptome was improved using additional RNA reads from reproductive tissues. Newly assembled contigs were annotated in the Gene Ontology database and were assigned to 3 main categories. The AP2/ERF superfamily is the third most represented compared with other transcription factor families. A comparison with genomic scaffolds led to an estimation of 114 *AP2/ERF* genes and 1 soloist in *Hevea brasiliensis*. Based on a phylogenetic analysis, functions were predicted for 26 *HbERF* genes. A relative transcript abundance analysis was performed by real-time RT-PCR in various tissues. Transcripts of ERFs from group I and VIII were very abundant in all tissues while those of group VII were highly accumulated in latex cells. Seven of the thirty-five ERF expression marker genes were highly expressed in latex. Subcellular localization and transactivation analyses suggested that *HbERF-VII* candidate genes encoded functional transcription factors.

## Introduction

Transcription factors (TFs) activate or repress the transcription of genes. The regulation of gene expression can be constitutive, tissue-specific or induced in response to environmental stimuli [1]. Plants are sessile organisms, which develop different mechanisms to protect themselves against aggressors, but also to adapt to various environments. In plants, this involves exogenous and endogenous signals, such as hormones. The gaseous plant hormone ethylene is reported to play an active role in a wide range of developmental and adaptation processes [2]–[4]. Studies on ethylene signalling have revealed a linear transduction pathway that leads to the activation of transcriptional regulators belonging to the Ethylene Response Factor (ERF) type. The Ethylene Response Factor (ERF) is one of the most important families of transcription factors and plays a key role in hormone, sugar and redox signalling in a context of abiotic and biotic stress [5]. ERFs have one AP2 domain, which is involved in DNA binding. This domain is about 60 amino acid residues which recognize GCC or DRE boxes in the promoter sequence of their target genes. Based on this conserved domain, ERFs were classed in ten groups by Nakano [6] or several subclasses by Sakuma [7]. The ERF family belongs to the AP2/ERF superfamily, which has been described for several species. Of the few woody plant species studied [8]–[10], *AP2/ERF* genes have been identified for only one subtropical crop: *Hevea brasiliensis* [11].

**Figure 1:**
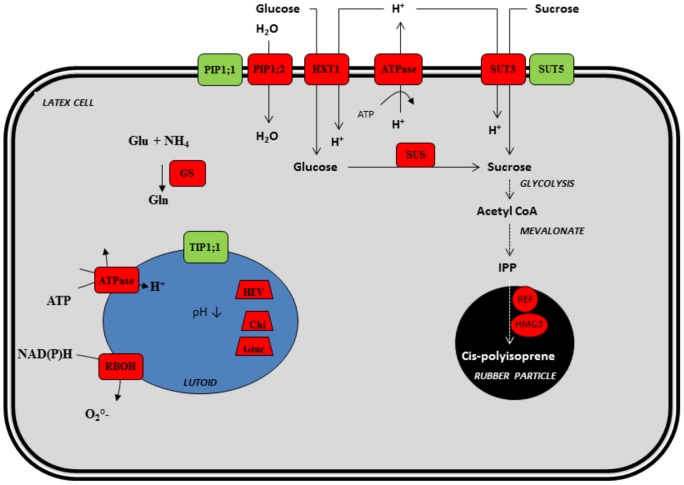
General scheme of ethylene-induced biochemical pathways in latex cells. Factors in red and green are activated and inhibited by ethephon or ethylene, respectively. Factors are: Chi (Chitinase), Glu (Glucanase), GS (Glutamine synthetase), HEV (Hevein), HMG (3-hydroxy-3-methylglutaryl-coenzyme A reductase), HXT (Hexose transporter), PIP (Plasma membrane intrinsic protein), RBOH (NADPH oxidase), REF (Rubber elongating factor), SUS (Sucrose synthase), SUT (Sucrose transporter).

*Hevea* is the only commercial source of natural rubber. Natural rubber is synthesized in the cytoplasm of laticifers, which are periodically emitted from the cambium [12]. A laticifer is a cellular network created by anastomosis of latex cells, which is embedded in phloem tissues. The latex is collected by tapping the soft bark tissues. Ethephon, an ethylene releaser, is applied to the surface of the tapping panel to stimulate latex production. Ethephon induces several biochemical processes in laticifers [13], such as sucrose loading, water uptake, nitrogen assimilation or synthesis of defence proteins, involving a large number of ethylene-response genes [14]–[20], whereas its direct role in rubber biosynthesis is controversial [21] (Figure 1). For that reason, ethylene biosynthesis and signalling pathways have been intensively studied in *Hevea* [14], [15], [17], [22]–[24]. Based on NGS sequencing of five tissue-type libraries (latex, bark, leaf, root, somatic embryogenic tissues), 173 AP2 domain-containing transcripts have been identified in *Hevea*, of which 142 have a full-length AP2 domain [11]. In *Hevea*, the ERF transcription factor family consists of 115 members divided into ten main groups. The three groups VII, VIII and IX account for more than 50% of HbERF. The expression of some *HbERF* genes has been associated with somatic embryogenesis [25], jasmonic acid-induced laticifer differentiation [26], and abiotic stress [27], [28]. According to Duan, the three *HbERF* genes induced upon laticifer differentiation correspond to three members of group VII (HbERF-VIIa3, HbERF-VIIa17 and HbERF-VIIa1) [11].

This paper first set out to more effectively estimate the number of *HbAP2/ERF* genes by completing the *Hevea* clone PB 260 transcriptome by sequencing additional tissues and annotating functions, particularly transcription factors, and comparing these RNA sequences with available genomic sequences. A reproductive tissue-type library (immature and mature male and female flowers, zygotic embryos) was sequenced and re-assembled with reads from previously sequenced libraries (leaf, bark, latex, root, somatic embryogenesis tissues) using the same bioinformatics pipeline published by Duan [11]. Then, a gene expression analysis was performed in various tissues using real-time RT-PCR in order to identify *AP2/ERF* genes potentially involved in the regulation of latex production. The function of members from group HbERF-VII, which are highly expressed in latex, was tested by subcellular localization and transactivation of an artificial GCC-box containing promoter.

## Materials and Methods

### Plant material

For the *Hevea* transcriptome analysis, plant material of clone PB 260 was grown according to the conditions described in Duan and coll. [11]. Latex, leaf, bark, and zygotic embryo RNA samples were collected and prepared at the Sembawa Centre, IRRI, P.O Box 1127, Palembang 30001, Indonesia (2 degrees 55 minutes 39.15 seconds South, 104 degrees 32 minutes 18.9 seconds East). Reproductive (immature and mature male and female flowers, zygotic embryos) RNA samples were collected and prepared at the CRRC, RRIT, Sanam Chaikhet District, Chachoengsao 24160, Thailand (13.39°N latitude and 101.26°E longitude). These locations and our activities did not require any specific permission. The field studies did not involve endangered or protected species.

For the relative transcript abundance analysis, the plant material was the same as for the transcriptome analysis, except latex and bark, which were harvested from 5-year-old trees without ethylene treatment and collected at IRRI's Sembawa Centre.

### Total RNA isolation

All samples were frozen in liquid nitrogen and stored in the freezer at −80°C pending total RNA extraction. Total RNAs were isolated using the caesium chloride cushion method adapted from Sambrook and coll. [29] by Duan and coll. [30]. One gram of fresh matter was ground and transferred to a tube containing 30 mL of extraction buffer consisting of 4 M guanidium isothiocyanate, 1% sarcosine, 1% polyvinylpyrrolidone and 1% β-mercapto-ethanol. After homogenization, tubes were kept on ice and then centrifuged at 10,000 g at 4°C for 30 min. The supernatant was transferred to a new tube containing 8 mL of 5.7 M CsCl. Ultracentrifugation in a swinging bucket was carried out at 89,705 g at 20°C for 20 h. The supernatant and caesium cushion were discarded whilst the RNA pellet was washed with 70% ethanol. After 30 min of air drying, the pellet was dissolved in 200 µL of sterile water. Although DNA could not cross the caesium cushion for this centrifugation condition, DNA contamination was checked by PCR amplification using primers of the Actin gene including the intron sequence. RNAs were stored at −80°C.

### Sequencing technique and contig assembly

Total RNA samples of each reproductive tissue were pooled together. Single-stranded cDNA was synthesised from pooled RNA samples. Pyrosequencing was carried out using a GS FLX (Roche/454) Titanium run (Roche Applied Science) by the GATC-Biotech company in Germany. A 454 sequencing half-run (200 Mbp) generated more than 500,000 reads with an average read length of 400 bp for each library according to the manufacturer. Reads were analysed using the ESTtik tool (Expressed Sequence Tag Treatment and investigation kit) [31] available on the Southgreen bioinformatics platform (www.southgreen.fr) and modified for the analysis of 454 data items. Reads were first cleaned to avoid mis-assembly by discarding sequences that were both lower than 120 bp and of low complexity. We then discarded non-coding reads by comparing the reads against the fRNAdb database using the Megablast algorithm with an e-value cutoff of 1e-20 [32]. More than 400,000 cleaned reads were obtained for this library. Reads from this library (Accession: PRJNA236464, ID: 236464) and those published by Duan and coll. (Accession: PRJNA235297 ID: 235297) were then assembled in contigs using the TGICL program [33], integrated in the ESTtik pipeline. The removal of poor end regions of reads and the computation of overlaps between reads was done using the default parameters of the CAP3 program (best hit cut-off for difference -b = 20; best hit for clipping –c = 12) [34]. Clustering was carried out for reads with an overlap of at least 60 bp and 94% identity between reads. The second step was an assembly of reads from each cluster with greater stringency: the length of sequence overlap was then 60 bp with 95% identity between reads. The transcript sequence database consisted of contigs.

The annotation of contigs was processed using Orfpredictor to obtain the longest ORFs leading to an Interproscan annotation. Then, all contigs were aligned to the NCBI non-redundant protein database using BLASTX to obtain an xml output format. Lastly, we treated the Interproscan and BLASTX results as the input files of the Blast2Go program and generated the output file listing the GO number for each annotated contig. By using this file, combining their GO databases (component.ontology, function.ontology, and process.ontology) downloaded from the GO website, we generated the figure using our own perl program.

### Primer design and analysis of transcript abundances by real-time RT-PCR

Several rules were applied in order to reduce the risk of error in relative gene expression data. The integrity of total RNA was checked by electrophoresis. Primers were designed at the 3′ side of each sequence in order to reduce the risk of error due to short cDNA synthesis using the Primer 3 module of Geneious (Biomatters Ltd., New Zealand). Real-time PCR amplification and the fusion curve were carried out using a mix of cDNAs in order to check the specificity of each pair of primers. Primer sequences are listed in Table S1 in File S2.

cDNAs were synthesized from 2 µg of total RNA to the final 20 µL reaction mixture using a RevertAidTM M-MuLV Reverse Transcriptase (RT) kit according to the manufacturer's instructions (MBI, Fermentas, Canada). Full-length cDNA synthesis was checked on each cDNA sample by PCR amplification of the Actin cDNA using primers at the cDNA ends. Quantitative gene expression analysis was finally carried out by real-time RT-PCR using a Light Cycler 480 (Roche, Switzerland). Real-time PCR reaction mixtures consisted of 2 µL RT product cDNA, 0.6 µL of 5 µM of each primer, and 3 µL 2× SYBR green PCR master mix (LightCycler® 480 SYBR Green I Master, Roche Applied Sciences) in a 6-µL volume. PCR cycling conditions comprised one denaturation cycle at 95°C for 5 min, followed by 45 amplification cycles (95°C for 20 s, 60°C for 15 s, and 72°C for 20s). Expression analysis was carried out in a 384-well plate. Samples were loaded using an automation workstation (Biomek NX, Beckman Coulter).

Real-time PCR was carried out for eleven housekeeping genes in order to select the most stable gene as the internal control for all the compared tissues (*HbelF1Aa*, *HbUBC4*, *HbUBC2b*, *HbYLS8*, *HbRH2b*, *HbRH8*, *HbUBC2a*, *HbalphaTub*, *Hb40S*, *HbUbi*, *HbActin*). *HbRH2b* was selected as the best reference gene due to its stability in tissues from immature and mature male and female flowers, zygotic embryos, latex and bark. The homogeneity of the *HbRH2b* gene Cp confirmed that it could be used as an internal reference gene (Table S2 in File S2). The *HbRH2b* gene was amplified in each reaction plate in parallel with target genes. The transcript abundance level for each gene was relatively quantified by normalization with the transcript abundance of the reference *HbRH2b* gene. Relative transcript abundance took into account primer efficiencies. All the normalized ratios corresponding to transcript accumulation were calculated automatically by Light Cycler Software version 1.5.0 provided by the manufacturer using the following calculation: Normalized Ratio = Efficiency ^−Δ*(Cp target-Cp RH2b)*^.

### Statistical data analyses

Real-time PCR reactions were set up with three biological replications. Statistical analysis was performed with an ANOVA after logarithmic transformation of raw data. The ANOVA was followed by a Student Newman-Keuls test when the values of relative transcript abundances were compared for immature and mature flowers, zygotic embryos, latex and bark. Values with the same letter did not differ significantly at the 0.05 probability level.

### Phylogenetic analysis of the AP2 domain from ERF marker genes

The full AP2-domain sequences derived from *Hevea*, *Arabidopsis*, *Populus*, *Vitis*, *Malus×domestica*, *Oryza* and *Zea mays* AP2-domain proteins of around 60 amino acids were then aligned using MUSCLE software [35], [36] which uses a progressive multiple alignment method. The alignment was curated by Gblocks software [37], searching for at least 10-amino-acid-long conserved blocks, and the block with 57 amino acids was extracted. This block of 57 amino acids was used to construct the phylogenetic tree using PhyML software [38], which implements a maximum likelihood tree reconstruction method, using the LG+gamma model, starting from a BioNJ tree [39]. A RAP-Green analysis was performed on the initial PhyML tree to improve gene function inferences and predict gene duplications in phylogenetic trees [40]. The final tree was drawn and displayed with the Archaeoptheryx program, and rooted on the branch separating the RAV family from the rest of the tree. Branch supports were computed using the aLRT-SHlike method [41].

### Cis-acting element analysis and determination of conserved motifs

*In silico* promoter analysis was searched again in the PLACE database [42] and The PlantCARE database [43]. The *Hevea* genomic scaffold was provided for the *HbERF-VIIa7*, *HbERF-VIIa17* and *HbERF-VIIa20* genes by the CATAS-BIG *Hevea* Genome Project coordinated by Prof Chaorong Tang and Prof Songnian Hu. A 2000 bp sequence upstream from the start codon was scanned for the presence of putative *cis*-acting regulatory elements using the database associated search tools. The number of copies for each *cis*-acting element was then counted. Conserved motifs were investigated by multiple alignment analyses using ClustalW and MEME version 3.0 [44].

### Subcellular localization and transcriptional activity tests by transient expression in a single cell system

Tobacco protoplasts were used in the subcellular localization because *Hevea* protoplasts have a short viability [45]. GFP C-terminal fusions were obtained with *HbERF-VIIa7*, *HbERF-VIIa17* and *HbERF-VIIa20* and used for tobacco protoplast BY-2 transfection according to Chaabouni and coll. [46]. The subcellular location of the fluorescence was determined after 20 hours using a DM4500 microscope (Leica).

A transactivation experiment was carried out according to the procedure published by both Chaabouni and Pirrello [46], [47]. A synthetic reporter construct (4XGCC-GFP) was used [47]. Effector constructs were generated by fusing the 35S promoter to the CDS of the genes (*HbERF-VIIa7*, *HbERF-VIIa17* and *HbERF-VIIa20*). For transient assays, tobacco (*Nicotiana tabacum*) BY-2 protoplasts were co-transformed with reporter and effector constructs [48]. Transformation assays were performed in three independent replicates. After 16 h, GFP expression was analysed and quantified by flow cytometry (FACS Calibur II instrument, BD Biosciences) on a flow cytometry platform (MRI). Data were analysed using Flowing software. For each sample, 100–400 protoplasts were gated on forward light scatter (FSC) and side light scatter (SSC) to check the size and the structure of protoplasts and eliminate debris from the analysis. The GFP fluorescent protoplasts used for the calculation of GFP activity were selected within a gate. This gate was previously defined by the comparison between transformed protoplasts (co-transformation with the effector plasmid lacking the *HbERF* coding sequence) and non-transformed protoplasts. The green fluorescence detected in the defined gate showed a marked hook. The GFP fluorescence per population of cells corresponded to the average fluorescence intensity of the cell population after subtraction of autofluorescence determined with non-transformed BY-2 protoplasts. The data were normalized using an experiment with protoplasts transformed with the reporter vector in combination with the vector used as the effector plasmid, but lacking the *HbERF* coding sequence.

## Results

### Functional annotation and classification of a comprehensive *Hevea* transcriptome

**Figure 2:**
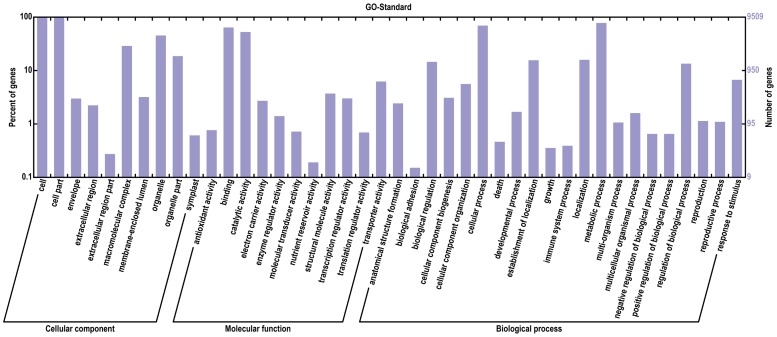
Gene Ontology classification of *Hevea brasiliensis* in various tissue types.

**Figure 3:**
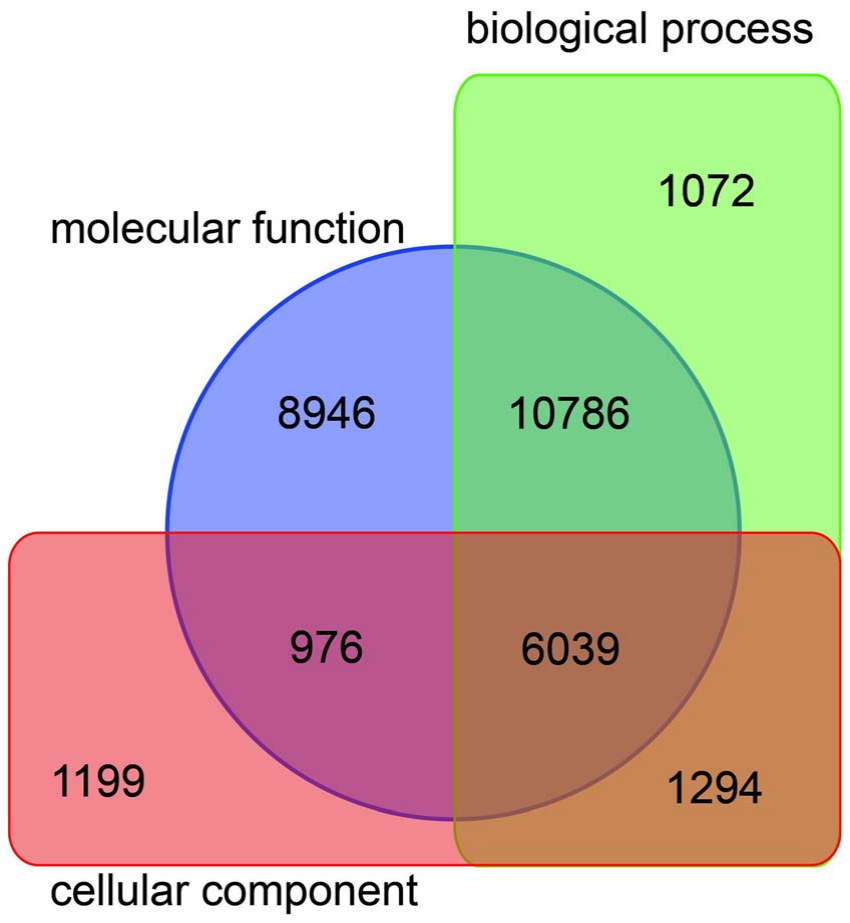
Venn diagram of the elements of the three GO terminologies.

Transcript sequences were previously produced from several tissue-type libraries (somatic embryogenic tissues, leaf, bark, latex and root) by the pyrosequencing GS-FLX 454 technique in order to have the most complete representation of the transcriptome of the *Hevea* clone PB 260 [11]. We supplemented this reference transcriptome with RNA sequences from a reproductive tissue-type library (immature and mature male and female flowers, zygotic embryos). All reads generated for these six libraries were re-assembled using the automatic pipeline described by Duan and coll. [11]. A total of 3,525,203 reads were cleaned and assembled in 86,941 contigs (Table S3 in File S2). From these contigs, 30,342 were then annotated in the Gene Ontology (GO) database, and 30,312 were classed using BLASTX (Table S4 in File S2). All contigs were categorized in 42 functional groups and classed in three ontologies: cellular component, molecular function and biological process (Figure 2). The largest number of contigs was assigned to the molecular function (26,747 contigs), which contained two major subcategories (binding and catalytic activity). This was followed by the biological process (19,191 contigs), which contained six major subcategories (biological regulation, cellular process, establishment of localization, localization, metabolic process, and regulation of biological process). The minority category was the cellular component (9,508 contigs), which contained five subcategories (cell, cell part, macromolecular complex, organelle and organelle part). Given that the elements of the three GO terminologies are not independent, a Venn diagram was drawn in order to illustrate their relationship (Figure 3).

**Figure 4:**
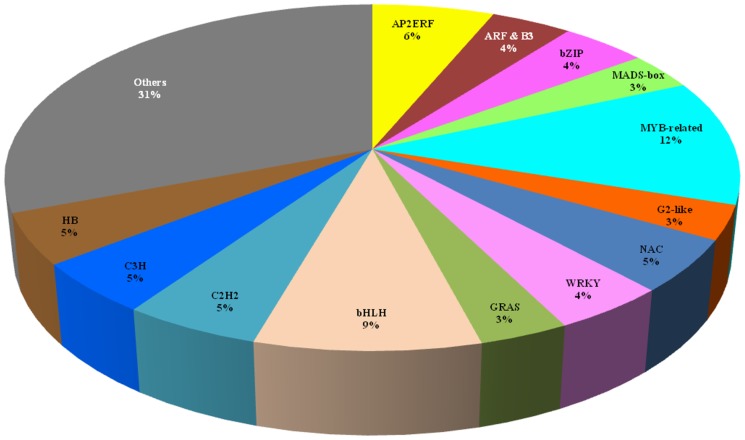
Transcription factor family distribution and percentage in *Hevea brasiliensis.*

The transcription regulator activity belonging to the molecular function category accounted for 2,448 contigs classed in 58 transcription factor families (Table S5 in File S2). Thirteen transcription factor families accounted for more than 3% of contigs (75 contigs) related to transcription factors (Figure 4). Among them, AP2/ERF, MYB, bHLH were the three largest families with at least 6% of contigs. The search for AP2 domain-containing genes in the genome database from the CATAS/BIG project showed 114 *AP2/ERF* genes found in this new transcriptome database from the rubber clone PB 260, which contains one soloist not found in the clone CATAS 7-33-97. This revealed better prediction than the Duan database counting 142 genes including 28 miss-assembled contigs [11] (Table S6 in File S2). Although most of the genomic scaffolds (109) harboured 1 gene only, 4 scaffolds had 2 genes (scaff38: HbERF-IIIb1 and HbERF-IIIc1; scaff419: HbERF-VIIIa12 and HbERF-VIIIa13; scaff 3809: HbERF-IIa3 and HbERF-IIb1; scaff6313: HbERF-Va2 and HbERF-Va4) and 1 scaffold contained 3 genes (scaff2511: HbERF-IXb1, HbERF-IXb2, HbERF-IXc1.2). The number of members of the *Hevea* AP2/ERF superfamily was compared with six other species (Table 1). *Hevea brasiliensis* showed the smallest number of *AP2/ERF* genes (114), followed by *Prunus persica* (131), *Arabidopsis thaliana* (147), *Vitis vinifera* (149), *Oryza sativa* (180) and *Populus trichocarpa* (202). This variation in gene number was mostly due to the number of genes from the ERF family, with some ERF groups being highly duplicated, such as groups II, III and IX.

**Table 1:** Number of AP2/ERF genes in plant species according to genomic analyses: *Arabidopsis thaliana*, *Oryza sativa*, *Solanum lycopersicum*, *Hevea brasiliensis*, *Populus trichocarpa*, *Prunus persica*, and *Vitis vinifera*.

Plant species		*Arabidopsis*	*Oryza*	*Solanum*	*Hevea*	*Populus*	*Prunus*	*Vitis*
Family	Group							
	I	10	9	ND	9	5	6	5
	II	15	16	ND	11	20	9	8
	III	23	27	ND	13	35	23	22
	IV	9	6	ND	5	6	7	5
	V	5	8	19	7	10	11	11
ERF	VI	8	6	4	6	11	3	5
	VI-L	4	3	3	3	4	4	2
	VII	5	15	5	6	6	6	3
	VIII	15	15	9	11	17	10	11
	IX	17	18	28	12	42	19	40
	X	8	12	9	8	9	6	10
	Xb-L	3	10	0	0	4	0	0
	Subtotal	122	145	-	87	169	104	122
AP2		18	29	18	20	26	21	20
RAV		6	5	3	3	6	5	6
Soloist		1	1	ND	0	1	1	1
Total		147	180	-	114	202	131	149

The ERF family has been classed according to Nakano's method. ND: not determined.

### Relative transcript abundance in various tissues

**Figure 5:**
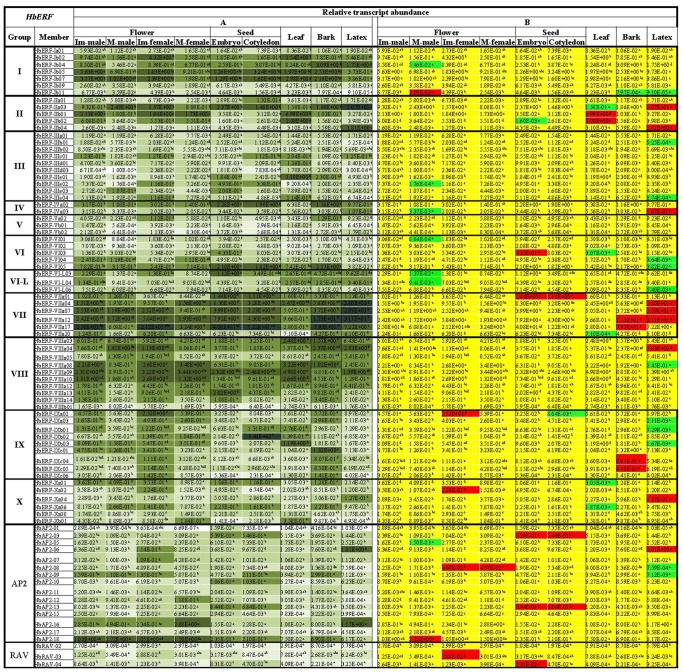
Relative transcript abundance profile of 107 *HbERF* genes for various tissue types (both immature and mature male and female flowers, zygotic embryos, leaf, bark and latex). The relative transcript abundances were measured by real-time RT-PCR. Values are the means of the relative transcript abundances of three biological replicates. (Figure 4A) heat map representation of the expression profile was used for values ranging as from dark (≥10) to light green (≤10^−5^). (Figure 4B) Values of relative transcript abundances in various tissue types were analysed with XLSTAT software after log transformation. The statistical analysis was performed with an ANOVA followed by the Student Newman-Keuls test. Values with significantly high relative transcript abundances shown in red and significantly low relative transcript abundances shown in green. The non-significant genes are shown in yellow.

The relative transcript abundance of 84 *AP2/ERF* genes was analysed in various tissues (both immature and mature male and female flowers, zygotic embryos, leaf, bark and latex) (Figure 5). For the 66 *ERF* genes, a high relative transcript abundance (value higher than one for the *HbERF*/*HbRH2b* ratio) was observed in all tissues for ERF members from groups I (4/7 genes), II (4/5 genes), VII (5/6 genes) and VIII (6/10 genes) (Figure 5A). In addition, a high relative transcript accumulation has been found for a few members of other ERF groups (*ERF-IIIe01*, *HbERF-VI05*, *HbERF-IXa02*, *HbERF-IXb02*, *HbERF-IXb03* and *HbERF-IXc01*). For the AP2 and RAV families, only *AP2-6*, *AP2-16* and *AP2-18* showed a similar or higher relative transcript abundance than the *HbRH2* internal control.

According to the statistical analysis, 44 *HbAP2/ERF* expression marker genes showed a significant higher or lower relative transcript abundance level in one tissue compared with other tissues (Figure 5B). In mature male flowers, seven genes (*HbERF-Ib04*, *HbERF-IIIe02*, *HbERF-IVa03*, *HbERF-VI01*, *HbERF-VI-L03*, *HbERF-VI-L04*, and *AP2-5*) showed a significant lower transcript accumulation and two genes (*HbER-Ib11*, *HbAP2-18*) a higher relative transcript accumulation compared with other tissues. In immature female flowers, 3 genes (*HbERF-IXa02*, *HbERF-Xa02* and *HbRAV-03*) showed a specific high transcript accumulation, whereas *HbAP2-08* was highly expressed in both immature and mature female flowers. In zygotic embryos, seven expression marker genes were identified. The *HbERF-VIIa01*, *HbAP2-03* and *HbAP2-13* genes showed high relative transcript abundance in both the embryo body and cotyledon tissues. Three other genes showed embryo body tissue-specific relative transcript abundance: low abundance for *HbERF-IIb02* and high abundance for *HbERF-VI03* and *HbRAV-04*. The relative transcript abundance of *HbERF-IXa02* was specifically lower in the cotyledon than in the other tissues. Seven ERF genes were identified as leaf expression marker genes. The relative transcript abundance was lower for 5 genes (*HbERF-IIa03*, *HbERF-VI03*, *HbERF-VIIa20*, *HbERF-Xa01* and *HbERF-Xa06*) compared with the other tissues. A high relative transcript abundance in leaf tissue was recorded for 2 genes (*HbERF-IIb01*, *HbERF-IIb02*). Three *ERF* genes (*HbERF-VIIa17*, *HbERF-IXc4* and *HbERF-IXc5*) showed a higher relative transcript abundance in bark compared with the other tissues. Interestingly, the largest number of specific expression marker genes (16 *ERF*s and 3 *AP2s*) was found in latex. Eight genes (*HbERF-IIa03*, *HbERF-IIb04*, *HbERF-IVa03*, *HbERF-VIIa04*, *HbERF-VIIa07*, *HbERF-VIIIa04*, *HbERF-Xa04* and *HbAP2-06*) and eleven genes (*HbERF-IIIb01*, *HbERF-IIIe04*, *HbERF-VI04*, *HbERF-VI05*, *HbERF-VI-L06*, *HbERF-VIIIa08*, *HbERF-IXa03*, *HbERF-IXb01*, *HbERF-IXb03*, *HbAP2-08* and *HbAP2-09*) revealed a higher and lower relative transcript abundance, respectively. Two additional expression marker genes showed a higher (*HbERF-VIIa12*) or lower (*HbERF-Ib11*) relative transcript abundance in both bark and latex compared with other tissues.

### Identification of putative functions for *HbAP2/ERF* genes

The phylogenetic tree was constructed using the deduced amino acid sequences of the AP2 domain in order to identify *Hevea* potential orthologs to *Arabidopsis* proteins using RAP-Green analysis (Figures S1–S11 in File S1; Tables S7–S8 in File S2). Among the 114 *HbAP2/ERF* genes, functions could be predicted for 29 genes (Table S8 in File S2). Sixteen of the 35 ERF expression marker genes showed significant orthology parameters with 11 *Arabidopsis* genes as follows: *HbERF-IIb02* with *ORA47*, *HbERF-IIIe02* with *TINY*, *HbERF-VI01* with *CRF2/TMO3*, *HbERF-VI-L03* with *CRF10*, *HbERF-VIIa07* and *HbERF-VIIa12* with *RAP2.12*, *HbERF-VIIa17* and *HbERF-VIIa20* with *AtEBP/RAP2.3/ERF72*, *HbERF-VIIIa04* with *AtERF3*, *HbERF-IXa03* with *AtERF1*, *HbERF-IXb03* with *ERF5*, *HbERF-IXc04* and *HbERF-IXc05* with *ERF1*, *HbERF-Xa01* and *HbERF-Xa02* with *RAP2.6L*, and finally *HbERF-Xa06* with *ERF110* (Table 2). The putative functions of these ortholog genes were related to the response to biotic and abiotic stress, hypoxia, regulation of cell proliferation or root initiation, and hormone signalling. For the AP2 genes, only the latex expression marker *HbAP2-06* gene is predicted to encode an ortholog of WRINKLED1.

**Table 2:** Identification of putative functions for expression marker genes based on a phylogenetic tree analysis with *Arabidopsis thaliana* using the deduced amino acid sequences of the AP2 domain for each gene (Figure S1–S10 in File S1; Tables S7–S8 in File S2).

Gene	Transcript accumulation	Phylogenetic analysis			
		Orthologous gene	Accession No.	Putative function	Reference
*HbERF-Ib04*	Low in mature male flower	-	At4g39780-Ib	-	-
*HbERF-Ib11*	High in mature male flower	-	At4g13620-Ib	-	-
*HbERF-IIa03*	High in latex	-	-	-	-
*HbERF-IIb01*	High in leaf	-	At1g19210-IIb	-	-
*HbERF-IIb02*	High in leaf	ORA47	At1g74930-IIb	Biotic and abiotic stress	[102]
		-	-	-	-
*HbERF-IIb04*	High in latex				
*HbERF-IIIb01*	Low in latex	-	At1g63040-IIIb	-	-
*HbERF-IIIe02*	Low in mature male flower	TINY	At5g25810-IIIe	Suppressed cell proliferation and exhibited pleiotropic effects	[103]
*HbERF-IIIe04*	Low in latex	-	At4g32800-IIIe	-	-
*HbERF-Iva03*	High in latex	-	-	-	-
*HbERF-VI01*	Low in mature male flower	CRF2/TMO3	At4g23750-VI	Related to root initiation at later embryonic stages	[73], [104]
*HbERF-VI03*	High in zygotic embryo	-	-	-	-
*HbERF-VI04*	Low in latex				
*HbERF-VI05*	Low in latex	-	-	-	-
*HbERF-VI-L03*	Low in mature male flower	CRF10	At1g68550.1-VI-L	Cytokinin signalling pathway	[105]
*HbERF-VI-L04*	Low in mature male flower	-	-	-	-
*HbERF-VI-L06*	Low in latex	-	-	-	-
*HbERF-VIIa01*	High in zygotic embryo and cotyledon	-	-	-	-
*HbERF-VIIa04*	High in latex	-	-	-	-
*HbERF-VIIa07*	High in latex	RAP2.12	At1g53910	Activates gene expression for hypoxia	[80], [91]
*HbERF-VIIa12*	High in bark and latex				
*HbERF-VIIa17*	High in bark	AtEBP/RAP2.3/ERF72	At3g16770-VIIa	Jasmonate and/or ethylene	[84]
*HbERF-VIIa20*	Low in leaf				
*HbERF-VIIIa04*	High in bark and latex	AtERF3	At1g50640-VIIIa	Repressors of GCC box-dependent transcription	[106]
*HbERF-VIIIa08*	Low in latex	-	-	-	-
*HbERF-IXa02*	High in immature female flower, low in cotyledon	-	-	-	-
*HbERF-IXa03*	Low in latex	AtERF1	At4g17500-IXc	Activators of GCC box–dependent transcription	[106]
*HbERF-IXb01*	Low in latex	-	-	-	-
*HbERF-IXb03*	Low in latex	ERF5	At5g47230-IXc	Activators of GCC box–dependent transcription	[106]
*HbERF-IXc04*	High in bark	ERF1			
*HbERF-IXc05*	High in bark				
*HbERF-Xa01*	Low in leaf	Rap2.6L	At5g13330 -Xa	Expression drought-responsive gene	-
*HbERF-Xa02*	High in immature female flower				[107]
*HbERF-Xa04*	High in latex	-	-	-	-
*HbERF-Xa06*	Low in leaf	ERF110	At5g50080-Xa	Regulation of bolting time	[108]

**Figure 6:**
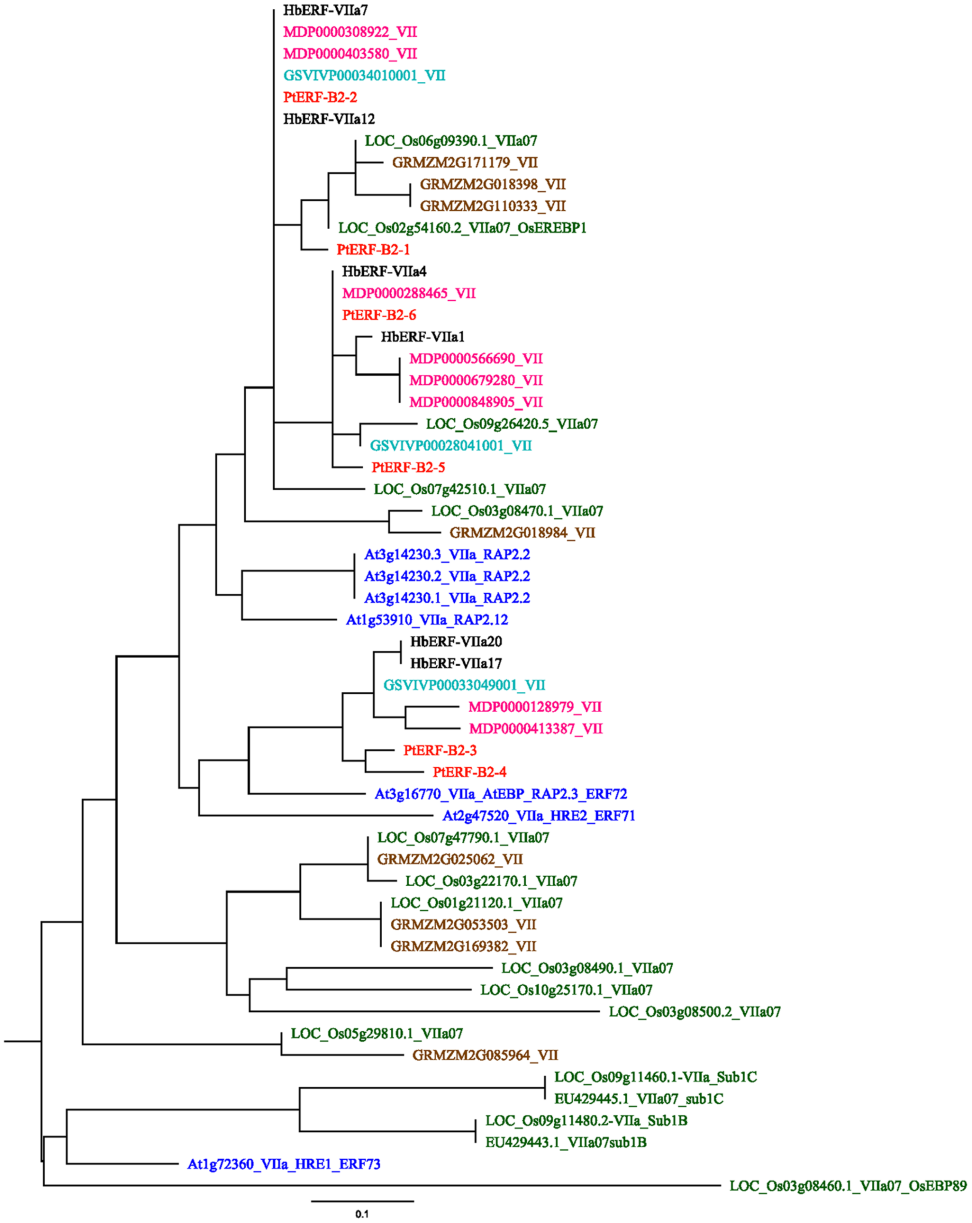
Phylogenetic tree illustrating the relatedness of group VII ERFs among plant species. The amino acid sequences of the AP2 domain were aligned using Muscle, and the phylogenetic tree was constructed using PhyML. The 7 plant species were: *Arabidopsis thaliana* (At2g47520,At3g16770, At1g72360, At1g53910, At3g14230.1,At3g14230.2, At3g14230.3); *Populus trichocarpa* (PtERF-B2-1, PtERF-B2-2, PtERF-B2-3, PtERF-B2-4, PtERF-B2-5, PtERF-B2-6) as named by Zhuang *et al*. (2008); *Vitis vinifera* (GSVIVP00034010001, GSVIVP00033049001, GSVIVP00028041001); *Oryza sativa* (Os02g54160.2, Os06g09390.1, Os03g08500.2, Os01g21120.1, Os03g22170.1, Os07g42510.1, Os07g47790.1, Os10g25170.1, Os03g08460.1, Os03g08490.1, Os09g26420.5, Os03g08470.1, Os05g29810.1, Os09g11460.1 and Os09g11480.2) as named by Nakano *et al*.(2006); *Orysa nivara* (EU429443.1submergence-1B,EU429445.1submergence-1C) as named by Fukao *et al*. (2009); *M.× domestica* (MDP0000848905, MDP0000679280, MDP0000566690, MDP0000413387, MDP0000403580, MDP0000308922, MDP0000288465 and MDP0000128979); *Zea mays* (GRMZM2G171179, GRMZM2G169382, GRMZM2G110333, GRMZM2G085964, GRMZM2G053503, GRMZM2G025062, GRMZM2G018984 and GRMZM2G018398) and *Hevea brasiliensis* (*HbERF-VIIa1* to *HbERF-VIIa23*) as named by Duan *et al*.(2013).

Transcripts of *ERFs* from group VII were highly accumulated in latex. Several members of this group were identified as key regulators of hypoxia-responsive genes in Arabidopsis but also in *Oryza* species. For that reason, an additional phylogenetic analysis was carried out using the 6 *Hevea* members, and 15 *Oryza sativa* and 2 *Oryza nivara* protein sequences, which have specific functions related to the response to submergence (Figure S11 in File S1). This phylogenetic analysis showed that the *HbERF-VIIa07* and *HbERF-VIIa12* genes were found to be orthologs to the *OsEREBP1*. Finally, a phylogenetic analysis was carried out by comparing AP2 domain-deduced amino acid sequences from five dicots (*Arabidopsis thaliana*, *Populus trichocarpa*, *Vitis vinifera*, *Malus×domestica* and *Hevea brasiliensis*) and two monocots (*Oryza sativa* and *Zea mays*) for group VII (Figure 6). The differentiation of members from ERF group VII clearly occurred before the speciation between dicot and monocot species because ERF-VIIs from all species were distributed in several clades [49]. Only one clade was early differentiated to generate genes involved in the tolerance of submergence in rice.

### Gene structure and in silico promoter analysis of *HbERF-VIIs*

**Figure 7:**
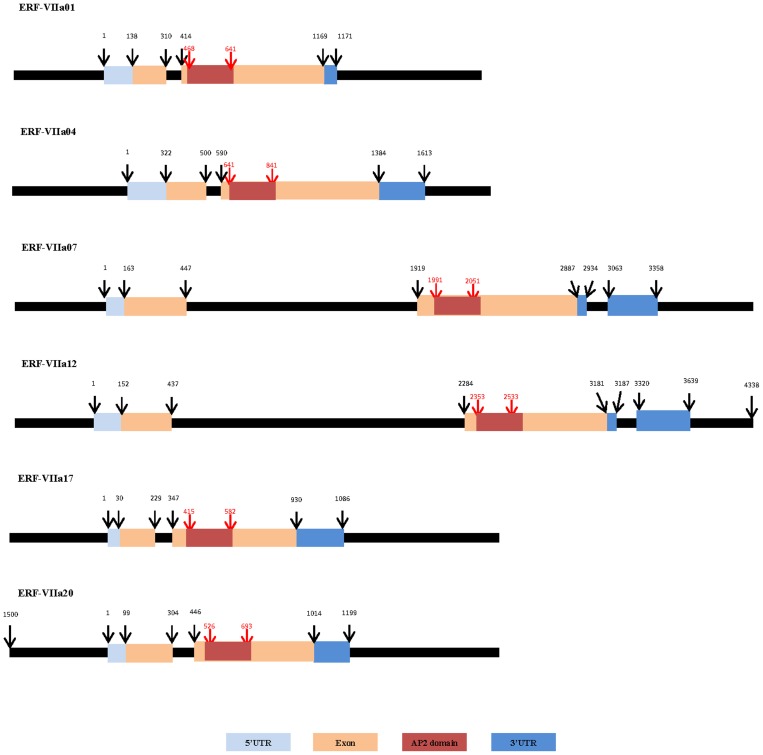
Gene structure of the 6 *HbERF-VII* genes.

Highly expressed in latex and putatively key regulators of hypoxia response, which is a phenomenon occurring in latex from TPD-affected trees, this study focused on gene structure and *in silico* promoter analysis of *HbERF-VIIs*. The gene structure of the 6 HbERF-VIIs was analysed using genomic scaffold sequences from the *Hevea* clone CATAS 7-33-97. These scaffolds revealed that the *ERF-VII* genes possessed 2 exons and at least 1 intron (Figure 7). The first exon was shorter (174–287 bp) than the second exon (568–968 bp), which contained the AP2 domain. Two genes (*HbERF-VIIa07* and *HbERF-VIIa12*) contained 2 introns, the second intron being located in the 3′UTR sequence. These two genes showed a large first intron (1472 to 1848 bp) compared with other genes (90–142 bp), and a short second intron (129–135 bp).

In order to better understand the regulation of these *ERFs*, an *in silico* analysis of the 2000 bp upstream sequence ATG codon of these scaffolds was carried out using PLACE and PlantCARE ([42], [43]; Table S9 in File S2). Forty-one *cis*-acting elements were selected for their putative role in the response to tissue specificity, hormones, sugar/starch and stress (Table 3). According to the selected regulatory groups, 11 *cis*-acting elements were observed. E-box, POLLEN1 and RAV1-A were found for all of the studied genes. Interestingly, CAT-box, meristem specific *cis*-acting elements only had a high frequency (27 copies) in *HbERF-VIIa1*. L1 box (L1 layer specific) and XYL (regulating secondary xylem development *cis*-acting element) only existed in *HbERF-VIIa17*. Thirteen *cis*-acting elements of six hormone responses (jasmonic acid, ethylene, abscisic acid, auxin, gibberellin and salicylic acid) were observed. Three *cis*-acting elements were found individually, TCA-element in *HbERF-VIIa20*, GCC-box in *HbERF-VIIa1*and JERE in *HbERF-VIIa17*. For sugar and starch responses, eight *cis*-acting elements (A-box, amylase box, CGACG element, CMSRE-1, OsBP-5, SRE, TATCCA element and TATCCAY motif) were found. The *cis*-acting elements in this regulatory function were distributed over the studied genes except *HbERF-VIIa7*. The majority of the upstream elements were categorized in stress-related responses. Eight *cis*-acting elements (ARE, DRE/CRT, LTRE, CNGTTR-motif, CANNTG-motif, TC-rich repeats, W-box and WUN-motif) were discovered. CANNTG-motif and TC-rich repeats were found for all of the studied genes, while the WUN-motif was specifically present in *HbERF-VIIa04*.

**Table 3:** *Cis-*acting regulatory elements identified in the *HbERF-VIIs* promoter region using 2000 bp region upstream ATG (start codon) by PLACE and PlantCARE.

Regulatory group	Cis-acting element	Function	Number of cis-acting elements					
			HbERF-VIIa01	HbERF-VIIa04	HbERF-VIIa07	HbERF-VIIa12	HbERF-VIIa17	HbERF-VIIa20
	(CA)n element	Embryo and endosperm-specific	-	-	-	1	1	2
	CAT-box	Meristem expression	27	1	-	-	-	-
	DRE2 core	Embryogenesis	-	-	-	-	-	1
Tissue-specific expression	E-box	Embryo and endosperm-specific	10	18	14	6	20	20
	L1 box	Layer L1 protoderm of organ primordia	-	-	-	-	1	-
	motif I	Root specific	-	-	1	-	1	-
	POLLEN1	Pollen specific expression	4	9	3	7	13	12
	RAV1-A	Tissue-specific expression	4	1	4	2	9	3
	RAV1-B	Tissue-specific expression	-	2	1	1	2	-
	TTAATGG-motif	Central cell-specific expression	-	1	1	-	1	-
	XYL	Regulating secondary xylem development	-	-	-	-	1	-
	ABRE	ABA signalling	-	-	-	-	7	2
	DPBF-1 and 2	ABA signalling and embryo-specification	4	-	-	1	6	2
	ARF	Auxin signalling	1	2	1	1	1	-
	TGA-element	Auxin signalling	1	-	-	-	1	-
	GARE-motif	Gibberellin signalling	-	-	-	1	1	2
Hormone response	TATCCAC box	Gibberellin response	-	2	-	-	2	-
	TCA-element	Salicylic acid signalling	-	-	-	-	-	2
	ERE	Primary ethylene signalling	-	1	1	-	-	-
	GCC-box	Ethylene signalling	1	-	-	-	-	-
	DRE/CRT	Ethylene signalling	3	4	-	-	1	2
	CGTCA-motif	Jasmonate signalling	-	-	-	-	3	4
	JERE	Jasmonate signalling	-	-	-	-	1	-
	T/G-box	Jasmonate signalling	2	1	-	-	1	-
	TGACG-motif	Jasmonate signalling	1	-	-	-	3	4
	A-box	Sugar repression	-	-	-	2	-	2
	amylase box	Sugar starvation	1	1	-	-	2	-
Sugar and starch response	CGACG element	Sugar starvation	-	1	-	-	-	2
	CMSRE-1	Sucrose-inducible expression	-	1	-	-	-	-
	OsBP-5	Starch synthase.	1	1	-	-	-	-
	SRE	Sugar repression	1	2	-	1	3	-
	TATCCA element	Mediates sugar and hormone regulation	3	4	-	-	7	1
	TATCCAY motif	Sugar repression	1	3	-	-	3	-
	ARE	Oxidative stress response	-	1	-	1	1	4
	ANAERO	Anaerobic gene regulation	1	5	-	2	4	3
	LTRE	Low temperature response	-	-	-	1	-	1
Stress-related response	CNGTTR-motif	MYB recognition site responses in stress and development	-	2	2	2	12	6
	CANNTG-motif	MYC recognition site responses in stress and development	10	20	14	6	20	20
	TC-rich repeats	Defence and stress responsiveness	2	3	2	1	2	1
	W-box	Multiple responses in stress and development	13	35	-	12	31	21
	WUN-motif	Wound-response element	-	1	-	-	–	-

### Function of *HbERF-VIIs*

**Figure 8:**
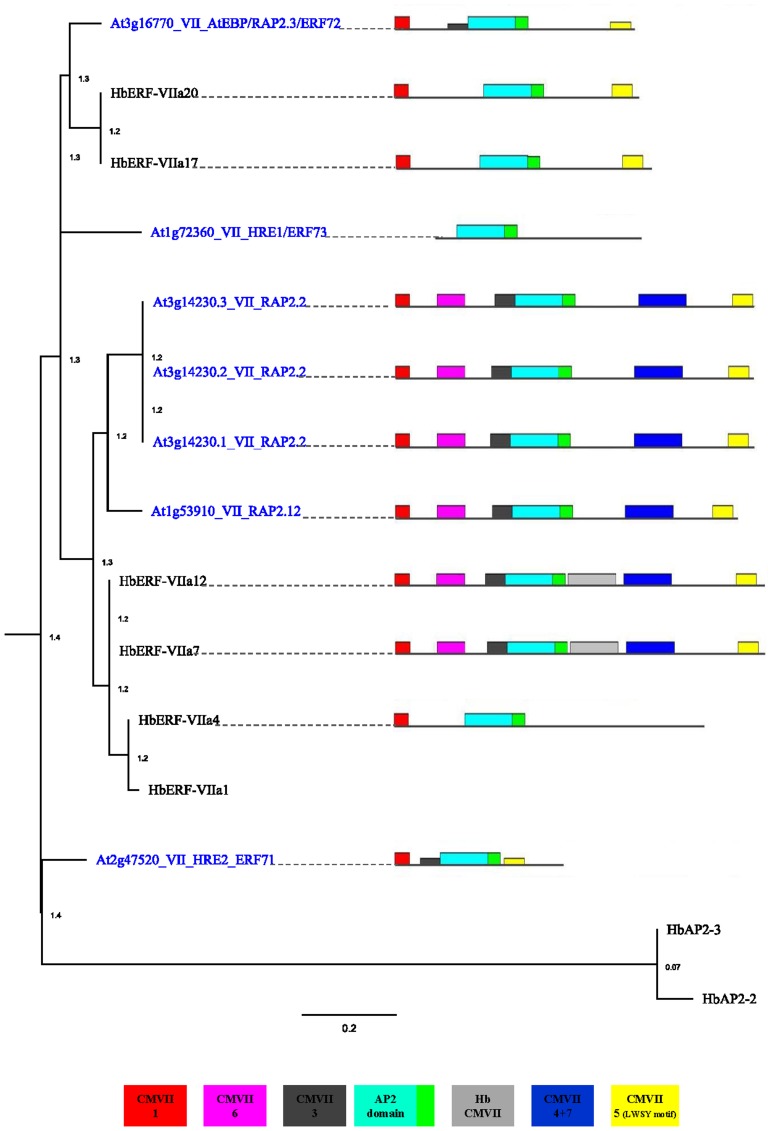
Phylogenetic tree and a schematic diagram of the conserved motif of group VII ERFs among *Arabidopsis thaliana* and *Hevea brasiliensis*.

A search for the conserved motifs outside the DNA-binding AP2 domain of the HbERF-VII deduced amino acid sequences was carried out using the MEME program [44], by comparison with *Arabidopsis* motifs (Figure 8; Table S10 in File S2). Interestingly, HbERF-VII proteins located in the same clades of the phylogenetic tree showed a similar pattern of motif distribution. Most of the *Arabidopsis* motifs (CMVII-1, CMVII-3, CMVII-4, CMVII-5, CMVII-6 and CMVII-7) existed in the HbERF-VIIs, except CMVII-2 and CMVII-8. One *Hevea*-specific motif (HbCMVII) with an unknown function was found in HbERF-VIIa07 and HbERF-VIIa12.

**Figure 9:**
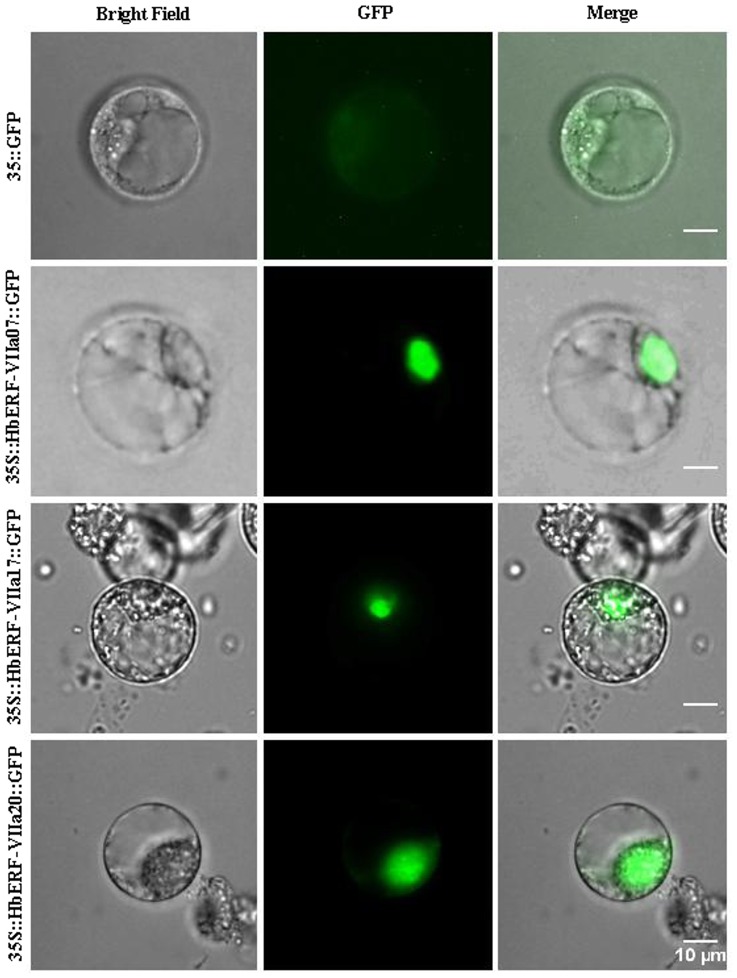
Subcellular localization of HbERF-VIIs. Tree HbERF-VII GFP fusion proteins (HbERF-VIIa7, HbERF-VIIa17 and HbERF-VIIa20) were transiently expressed in protoplasts from BY-2 tobacco cells under the control of the 35S promoter. Subcellular localization was analysed by confocal laser scanning microscopy. The merged pictures of the green fluorescence channel (middle panels) and the corresponding bright field (left panels) are shown (right panels). Control cells expressing fluorescence absence are shown in the top panel. The scale bar indicates 10 µm.

**Figure 10:**
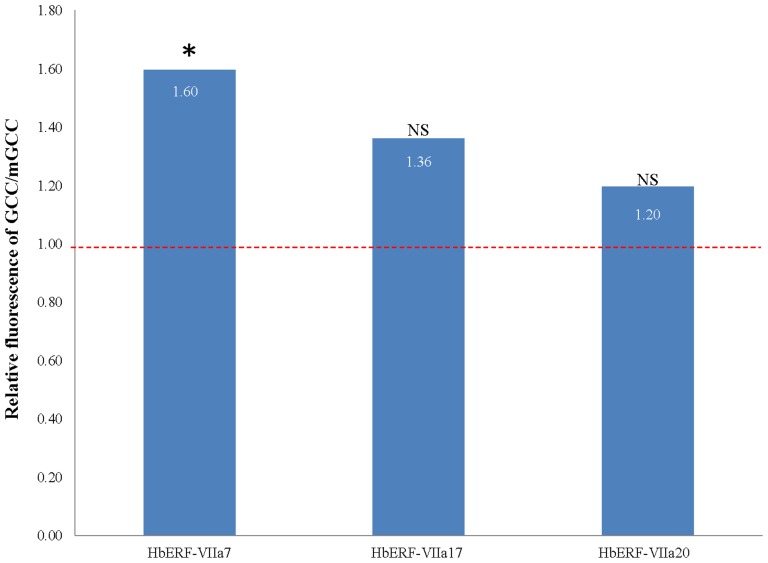
Transactivation of the synthetic GCC-box and mGCC containing promoters by HbERF-VIIa7, HbERF-VIIa17 and HbERF-VIIa20 proteins. Transient expression in BY-2 tobacco protoplasts co-transformed by pMDC32 harbouring the effector construct (ERF candidate genes under the control of the CaMV 35S promoter) and the reporter constructs, 4× GCC::GFP or 4× mGCC::GFP, respectively. Fluorescence activity was measured by flow cytometry for three independent biological replicates. The ratio of fluorescence between the constructs with functional GCC and mutated mGCC boxes revealed that ERF candidates are activators (ratio >1) or repressors (ratio <1). (*) indicates significant difference for the Student test (p<0.01).

In order to validate the function of HbERF-VIIs as transcription factors, a subcellular localization experiment was carried out using HbERF coding sequences/GFP translational fusion into pMDC83 (Figure 9). Transient expression into BY-2 tobacco protoplasts revealed GFP activity of the fusion protein in the nucleus for each tested HbERF, in contrast with a pMDC83 empty control plasmid. As previously demonstrated, transient transformation of protoplasts is a powerful tool for analysing the activity of transcription factors [47], [50]. The GFP reporter gene under the control of a synthetic promoter harbouring the GCC box *cis*-acting element was transactivated by the three HbERF-VII candidates (Figure 10). The effector constructs and the reporter constructs driven either by a GCC-rich **s**ynthetic promoter (GCC) or a synthetic promoter containing a mutated GCC motif (mGCC::TCCTCC) were co-transformed with 35S::HbERF-VII constructs into BY2 tobacco protoplasts. GFP activity was quantified by flow cytometry for at least 100 protoplasts in 6 biological replicates. The ratio of GFP activities between GCC::GFP and mGCC::GFP constructs revealed the capacity of HbERF-VII to activate (ratio >1) or repress (ratio <1) the GCC promoter. All three ERF-VIIs showed a ratio higher than 1 but only HbERF-VIIa07 could significantly be considered as activators.

## Discussion

### Towards a comprehensive *Hevea* transcriptome

Over the last decade, several studies were conducted on *Hevea brasiliensis* to identify ESTs from latex [51], [52], especially those involved in rubber biosynthesis [53], [54], and also TPD-related genes [55]. More recently, several high-throughput analyses have led to the identification of microRNAs [56], [57], transcriptomes [11], [58]–[60], and genomes [61] using NGS technologies. All the transcriptome analyses have provided a partial overview of the *Hevea* transcriptome, as RNAs from only a few specific tissues were sequenced, such as shoot apex [58] and leaf [61] from clone RRIM 600, along with leaf and latex from CATAS 7-33-97 [59]. This latter work by Xia and coll. also revealed incomplete sequences for the ERF family, with partial sequences unfit for classification [11]. Duan and coll. reported having developed a comprehensive transcriptome using several tissues (root, bark, latex, leaf, and tissues from somatic embryogenesis) from plants growing under different environmental conditions [11]. Our study improved this previous transcriptome by adding RNA sequences from reproductive tissues (immature and mature male and female flowers, zygotic embryos) and by performing gene annotation. Fifty-eight transcription factor families were identified accounting for 2448 contigs (8.07% of predicted genes). Another study based on the *Hevea* genome sequence showed 5978 transcription factors (8.5% of gene models) classed in 50 families [61]. This number of transcription factors is very high (2.5 to 6 times) compared with other species and even with our own annotation. This large number could be explained by an overestimation. For instance, based on repeat motif identification, Rahman and coll. found 139 *AP2* genes, 246 *ERF* genes and 25 *RAV* genes. The presence of the AP2 domain in three transcription factor families (AP2, ERF, RAV), and the B3 domains in two other families (RAV, B3) probably led to one contig being annotated in more than one family carrying the same specific domain. The careful annotation carried out in our work is likely to provide a more accurate estimation of transcription factors and the ERF family in particular, as in our annotation 58 transcription factor families are commonly found in 49 species [62]. The number of transcription factors ranged from 1291 to 3546 genes with, for instance, 2201 genes for *Manihot esculenta*, a Euphorbiaceae close to *Hevea brasiliensis*, and 2585 genes in *Populus trichocarpa* in woody species.

This study led to a better estimation of *AP2/ERF* genes for the *Hevea brasiliensis* clone PB 260. The present transcriptome database, supplemented with RNA sequences from reproductive tissues, contains a smaller number of *AP2/ERF* genes (114+1 soloist instead of 142 found in Duan's paper) closed to the genomic information provided by the Chinese *Hevea* Genome Project on the *Hevea* clone CATAS 7-33-97. Although the first assembly of RNA reads was carried out with 2,387,930 reads from GS-FLX 454 sequencing, the addition of 643,113 reads from the reproductive tissues in this study led to a significant improvement in sequence quality comparable with genomic data (Table S3 in File S2). The decrease in gene number between Duan and coll. and the present report is mostly due to the reduction in gene number for the ERF groups VII (from 23 to 6), VIII (from 15 to 11) and IX (from 19 to 12). This reveals that transcriptome analyses can be used to identify genes harboured in a plant genome but require RNA sequencing of large numbers of tissues from plants at different stages growing under various environmental conditions in order to cover all types of expression patterns.

### Some *HbAP2/ERFs* could play an important role in latex

Several members of three ERF groups (I, VII, VIII) are highly expressed in latex. In total, sixteen latex expression marker genes with higher or lower transcript abundance compared with other tissues were found in all ERF groups except group V. Seven highly expressed *ERFs* in latex, not significantly different from other tissues, belonged to groups II, IV, VII and VIII. HbERF-II and HbERF-VIIIa deduced proteins contained the repressor EAR motif. Their high expression under normal conditions suggests a certain negative control of ethylene response, especially in latex, by HbERF-IIa03 and HbERF-IIb04, and in leaves by HbERF-IIb01 and HbERF-IIb02. Members of group VIII are known to drive the response to jasmonate. This hormone is assumed to play a crucial role in the response to tapping and, in particular, to mechanical wounding of bark. The latex expression marker gene *HbERF-VIIIa04* had AtERF3, a repressor of abiotic stress response and an inducer of cell death, as an ortholog.

The HbERF-IVs correspond to DREB2 in Sakuma's classification. These genes are generally involved in the response to dehydration. The role of HbERF-IVa03 in the response to water stress is consistent since laticifers are subjected to recurrent osmotic stress after tapping and consequent latex flow. Group VII was described as a regulator of hypoxia-responsive genes, which will be developed in the next part of the discussion. For group X, one member was described as a master regulator of redox potential (RRTF1) [63]. In *Hevea*, *HbERF-Xb1* is ortholog to *RRTF1*but is not an expression marker gene. By contrast, the marker *HbERF-Xa04* did not have any known function.

With regard to the nine latex expression marker *ERF* genes with very low relative transcript abundance, two belonged to group III. This group corresponds to CBF/DREB1 in Sakuma's classification. In addition to low expression of the *HbERF-III* genes, this group III included a small number of genes in *Hevea* compared with other species. This could explain the low adaptation of this subtropical species to cold. Indeed, genetic analysis showed that a duplication of *CBF* genes was related to low temperature tolerance in *Arabidopsis*, tomato [64], maize [65], and *Eucalyptus* [66]. In wheat and barley, the Frost resistance-2 (Fr-2) locus is coincident with a cluster of more than 12 *CBF* genes [67]. In *Eucalyptus*, 14 of the 17 *CBF* genes are located in a cluster on chromosome 1 [68]. QTLs of cold tolerance have been linked to *CBF* genes in *Arabidopsis* [69], and *Eucalyptus nitens* [70]. Nevertheless, a general correlation between level of expression of *CBFs* and latitudinal position has been demonstrated [71] it has also been shown that the correlation between level of expression and level of cold tolerance is not so strict and in some accessions it is clearly dissociated [72]. Three other latex expression marker genes belong to ERF groups VI and VI-like, which are involved in the response to cytokinin [73]. Lastly, two latex expression marker genes belonging to group IX had orthologs with functions related to vascular cell division (HbERF-IXa03/AtERF99) [74], and programmed cell death (HbERF-IXb03/AtERF102) [75].

The last latex expression marker gene *HbAP2-6* encodes an ortholog of WRINKLED1 (WRI1). WRI transcription factors orchestrate tissue-specific regulation of fatty acid biosynthesis and in particular WRI1 is involved in seed storage metabolism in *Arabidopsis* [76], [77]. Glycolipids represented 27–37% of total lipids in *Hevea* latex [78]. This unusual glycolipid composition was suggested to be linked to the peculiar nature of this specialized cytoplasm expelled from the laticiferous system during tapping. The high relative transcript abundance of *HbAP2-6* could thus be related to the induction of genes involved in fatty acid biosynthesis in *Hevea*.

### *HbERF-VII* genes could play a role in hypoxia response

The ERFs from group VII are known to regulate hypoxia-responsive genes. ERF-VII proteins govern the response to low oxygen in plants by post-translational regulation. The regulation of these transcription factors has been characterized in depth for AtERF75/RAP2.2, AtEBP/RAP2.3, AtERF74/RAP2.12, AtERF71/HRE2 and AtERF73/HRE1 in *Arabidopsis* [79]–[82]. AtEBP also confers resistance to hydrogen peroxide and heat treatments [83], and this protein has been shown to interact with ACBP4 [84]. The stability of all these proteins is enabled by binding to the Acyl CoA binding proteins (ACBP). Under hypoxia, ERF-VII proteins are dissociated and then activate hypoxia-responsive genes. When normoxia is recovered, the N-end rule pathway is involved in the proteosomal degradation of ERF-VII [85]. The N-terminal MCGGAII motif of ERF-VII proteins is targeted by the N-end rule pathway associated with the proteosomal degradation pathway [85], [86]. The presence of the N-terminal MCGGAII motif in all the identified HbERF-VII deduced proteins, as in other species, suggests similar post-translational regulation in *Hevea*.

Most species have 3 to 8 *ERF-VII* genes: 5 for *Arabidopsis thaliana* [6], 6 for *Populus trichocarpa* [9], 3 for *Vitis vinifera* [8], 8 for *Malus×domestica* [87], 4 for *Solanum lycopersicon* [47], and 6 for *Hevea brasiliensis* (present work). Only rice has a larger number (15) of genes [6]. Interestingly, phylogenetic analysis of ERF group VII proteins revealed that *Oryza sativa* had one additional clade with three genes (*OsSUB1A*, *OsSUB1B*, *OsSUB1C*) [88]. Interestingly, *OsSUB1A* is involved in submergence tolerance [89], [90]. Thus, ERF-VIIs are pivotal regulators of responses to flooding and low oxygen [91]. These ERF-VII proteins have also been shown to be regulated by oxidative stress [92], [93]. The divergence of these genes clearly led to a specific adaptation of rice to submergence by orchestrating various acclimatization responses [94], by creating a rice-specific group consisting of Sub1 and Snorkel proteins [49]. By contrast, the high expression of ERF-VIIs in *Hevea brasiliensis* with a small number of genes suggests a transcriptional-based adaptation in latex cells.

Some hypoxia-responsive genes targeted by ERF-VIIs identified in *Arabidopsis* were also present in latex. An analysis of the gene expression pattern revealed that *Arabidopsis* RAP2.2 controls the induction of genes involved in sugar metabolism and fermentation pathway enzymes [95]. Alcohol dehydrogenase (ADH), non-symbiotic haemoglobin (HB1), SUCROSE SYNTHASE1 (SUS1) and SUCROSE SYNTHASE4 (SUS4) are considered as good markers of anaerobic response [49]. In order to overcome insufficient production of ATP by mitochondrial respiration, the catabolism of soluble sugars and starch can lead to an adjustment of the energy crisis by maintaining ATP production and NAD^+^ regeneration [79], [91], [95]. Fermentation of pyruvate to ethanol by pyruvate decarboxylase and ADH plays a central role in hypoxia response. *ADH* expression is actively induced by RAP2.12 in *Arabidopsis* [96]. In *Hevea*, *ADH*, *SUS1*, *SUS4* and *HB1* were transcribed in all tissues and especially in latex, with the *ADH* presence of two contigs (CL1Contig11114, CL1536Contig2), *HB1* presence of one contig (CL2139Contig4), and *SUS1* presence of four contigs (CL1Contig20718, CL33Contig19, CL33Contig3 and CL33Contig8) (Table S11 in File S2). Their corresponding enzyme activities had also long been recorded in latex [97]. The presence of *cis*-acting elements involved in anaerobic response (ANAERO) and the sugar or starch content (amylase box, CGACG, BP-5, TATCCAY) in the promoter sequence of four *HbERF-VII* genes also revealed potential transcriptional regulation by the metabolism.

### Response to low oxygen concentration in latex and regulation of rubber production

Sucrose is the source of carbon and energy for the biosynthesis of natural rubber. AcetylCoA generated by glycolysis is used by the mevalonate pathway to produce isopentenyl pyrophosphate (IPP). IPP is the precursor for elongation of the polyisoprene chain in rubber particles. Latex regeneration after tapping requires a lot of energy from glycolysis. A hypoxic condition was suggested in laticifers since the fermentative pyruvate metabolism is the main route of sugar degradation in latex cytosol [98]. The intermediate product, pyruvate, can be used by both aerobic and anaerobic pathways to generate acetyl CoA [97]. In addition, oxygen consumption has been observed for two latex-specific organelles, in the lutoids by peroxidase and NADH-quinone reductase, and the Frey-Wyssling particles by *o*-diphenoloxidase [97]. Lutoidic NAD(P)H oxidase generates toxic forms of oxygen such as superoxide anions, which are involved in lipid peroxidation of lutoids. Coagulant factors are released from the damaged lutoids and lead to the aggregation of rubber particles [99]. This *in situ* coagulation of rubber particles reduces latex flow after tapping. This physiological syndrome is called Tapping Panel Dryness (TPD). In TPD-affected trees, the consumption of oxygen by NADH-Cytochrome-c-oxidoreductase from lutoids is particularly high [100]. According to these authors, NADH-dependent consumption of oxygen is inhibited by superoxide dismutase activity [101].

The involvement of oxygen in the latex metabolism and TPD syndrome and the tolerance of rubber trees to wounding suggest that some HbERF-VIIs might play an important role in latex production. Indeed, three (*HbERF-VIIa04*, *HbERF-VIIa07*, *HbERF-*VIIa12) of the six *HbERF- VIIs* identified in this study as expression marker genes are highly regulated in latex and are orthologs to AtEBP/RAP2.3 and AtERF74/RAP2.12. Another *HbERF-VII* gene, *HbERF-VIIa17* ortholog genes to *AtEBP*, might play a role in the response to the accumulation of reactive oxygen species generated during latex regeneration. In addition, three *HbERF* genes induced upon laticifer differentiation [26], correspond to three members of group VII (HbERF-VIIa3, HbERF-VIIa17 and HbERF-VIIa1) according to Duan and coll. [11]. In the present work, we showed that HbERF-VIIa3 corresponds to the new HbERF-VIIa4. Further characterization of the *HbERF-VII* genes and their target genes should lead to the identification of new functions in *Hevea brasiliensis*.

## Supporting Information

File S1 Contains Figure S1, Phylogenetic tree of ERF group I. The deduced amino acid sequences of the AP2 domain from *Hevea* (black letter) and *Arabidopsis* (blue letter) were aligned using Muscle, and the phylogenetic tree was constructed using PhyML with an LG+T model. Expression marker genes are indicated in bold letters. Figure S2, Phylogenetic tree of ERF group II. The deduced amino acid sequences of the AP2 domain from *Hevea* (black letter) and *Arabidopsis* (blue letter) were aligned using Muscle, and the phylogenetic tree was constructed using PhyML with an LG+T model. Expression marker genes are indicated in bold letters. Figure S3, Phylogenetic tree of ERF group III. The deduced amino acid sequences of the AP2 domain from *Hevea* (black letter) and *Arabidopsis* (blue letter) were aligned using Muscle, and the phylogenetic tree was constructed using PhyML with an LG+T model. Expression marker genes are indicated in bold letters. Figure S4, Phylogenetic tree of ERF group IV. The deduced amino acid sequences of the AP2 domain from *Hevea* (black letter) and *Arabidopsis* (blue letter) were aligned using Muscle, and the phylogenetic tree was constructed using PhyML with an LG+T model. Expression marker genes are indicated in bold letters. Figure S5, Phylogenetic tree of ERF group VI. The deduced amino acid sequences of the AP2 domain from *Hevea* (black letter) and *Arabidopsis* (blue letter) were aligned using Muscle, and the phylogenetic tree was constructed using PhyML with an LG+T model. Expression marker genes are indicated in bold letters. Figure S6, Phylogenetic tree of ERF group VI-L. The deduced amino acid sequences of the AP2 domain from *Hevea* (black letter) and *Arabidopsis* (blue letter) were aligned using Muscle, and the phylogenetic tree was constructed using PhyML with an LG+T model. Expression marker genes are indicated in bold letters. Figure S7, Phylogenetic tree of ERF group VII. The deduced amino acid sequences of the AP2 domain from *Hevea* (black letter) and *Arabidopsis* (blue letter) were aligned using Muscle, and the phylogenetic tree was constructed using PhyML with an LG+T model. Expression marker genes are indicated in bold letters. Figure S8, Phylogenetic tree of ERF group VIII. The deduced amino acid sequences of the AP2 domain from *Hevea* (black letter) and *Arabidopsis* (blue letter) were aligned using Muscle, and the phylogenetic tree was constructed using PhyML with an LG+T model. Expression marker genes are indicated in bold letters. Figure S9, Phylogenetic tree of ERF group IX. The deduced amino acid sequences of the AP2 domain from *Hevea* (black letter) and *Arabidopsis* (blue letter) were aligned using Muscle, and the phylogenetic tree was constructed using PhyML with an LG+T model. Expression marker genes are indicated in bold letters. Figure S10, Phylogenetic tree of ERF group X. The deduced amino acid sequences of the AP2 domain from *Hevea* (black letter) and *Arabidopsis* (blue letter) were aligned using Muscle, and the phylogenetic tree was constructed using PhyML with an LG+T model. Expression marker genes are indicated in bold letters. Figure S11, Phylogenetic analysis of ERF group VII using the 6 *Hevea brasiliensis*, 15 *Oryza sativa* and 2 *Oryza nivara* members. The deduced amino acid sequences of the AP2 domain from *Hevea* (black letter) and *Oryza sativa* and *Oryza nivara* (green letter) were aligned using Muscle, and the phylogenetic tree was constructed using PhyML with an LG+T model.
(PDF)

File S2 Contains Table S1, List of primer sequences for 84 *AP2/ERF* genes and 11 housekeeping genes, and expected length of amplicons after amplification by real-time PCR in *H. brasiliensis* clone PB 260. Table S2, Comparison of Cp values, standard deviation and coefficient of variance for gene expression analysis by real-time RT-PCR of 11 housekeeping genes in 11 tissues from male and female immature and mature flowers, zygotic embryos, latex and bark. Table S3, Summary of 454 sequencing and clustering using automatic pipeline for various tissue-type libraries (embryogenic tissues, leaf, bark, latex, roots and reproductive tissue) and a global library combining reads from all tissues. Table S4, Annotation and classification of gene function according to GO analysis. Table S5, Classification of transcription factor family in *Hevea*. Table S6, Comparison between contigs generated by RNAseq (Duan et al. 2013 and this report) and genomic scaffold. Table S7, RAP-Green analysis for ortholog and paralog inferences in phylogenetic comparison between *Arabidopsis* and *Hevea*. Table S8, Identification of putative orthologs of *Hevea brasiliensis* AP2/ERFs using *Arabidopsis* TAIR database. Table S9, *Cis*-acting regulatory elements identified in HbERF-VIIs promoter region using −2000 bp region upstream ATG (start codon) by PLACE and PlantCARE. Table S10, Comparison of conserved motifs between *Arabidopsis* and *Hevea*. Table S11, Read distribution list corresponding to anaerobic-responsive genes.
(XLSX)
