# Supplementary figures and images for: Sequence and Expression Analyses of Ethylene Response Factors Highly Expressed in Latex Cells from Hevea brasiliensis
Source: PLoS One. 2014 Jun 27;9(6):e99367. doi: 10.1371/journal.pone.0099367 (PMC4074046; doi:10.1371/journal.pone.0099367)

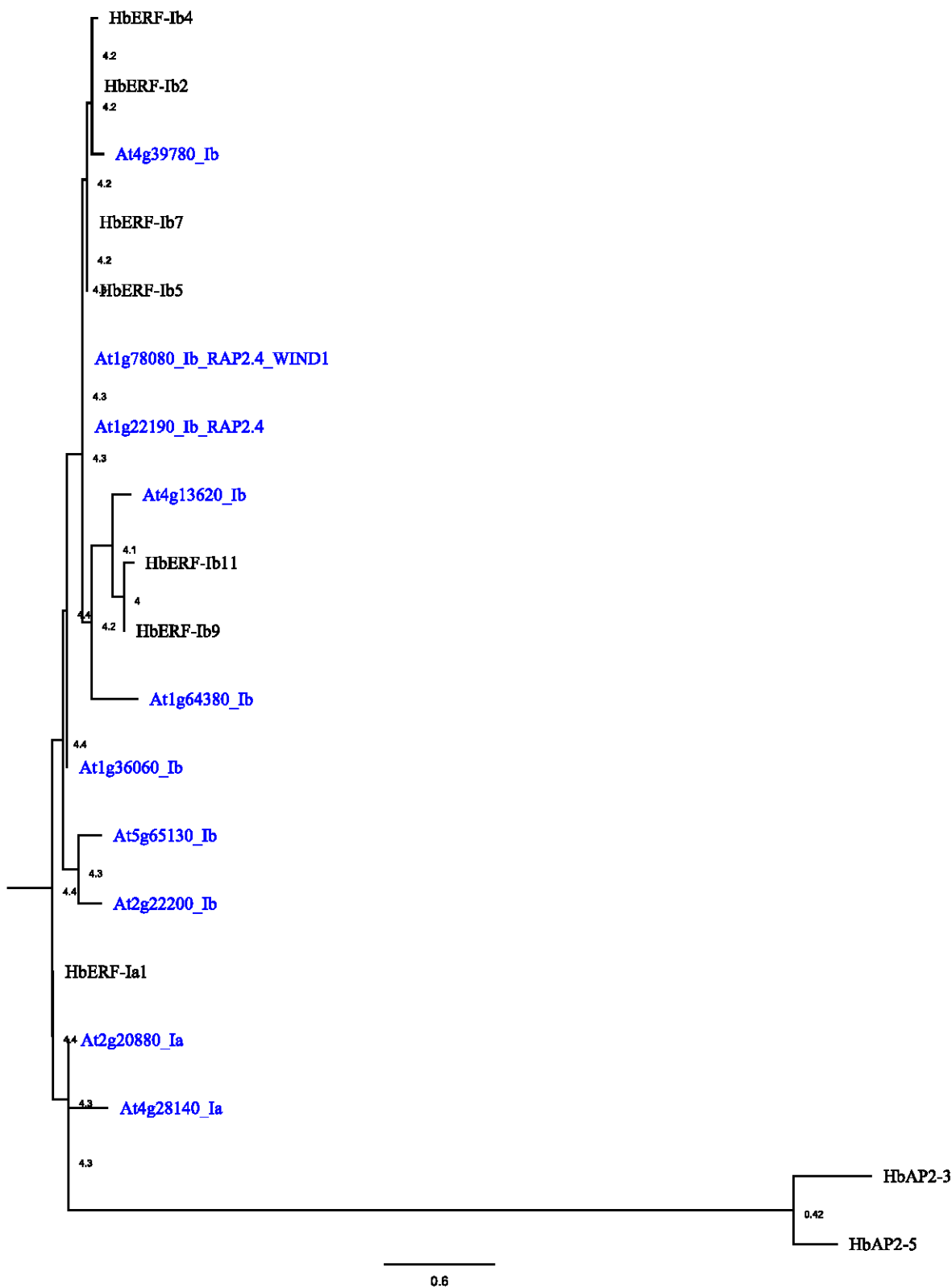

Figure 1

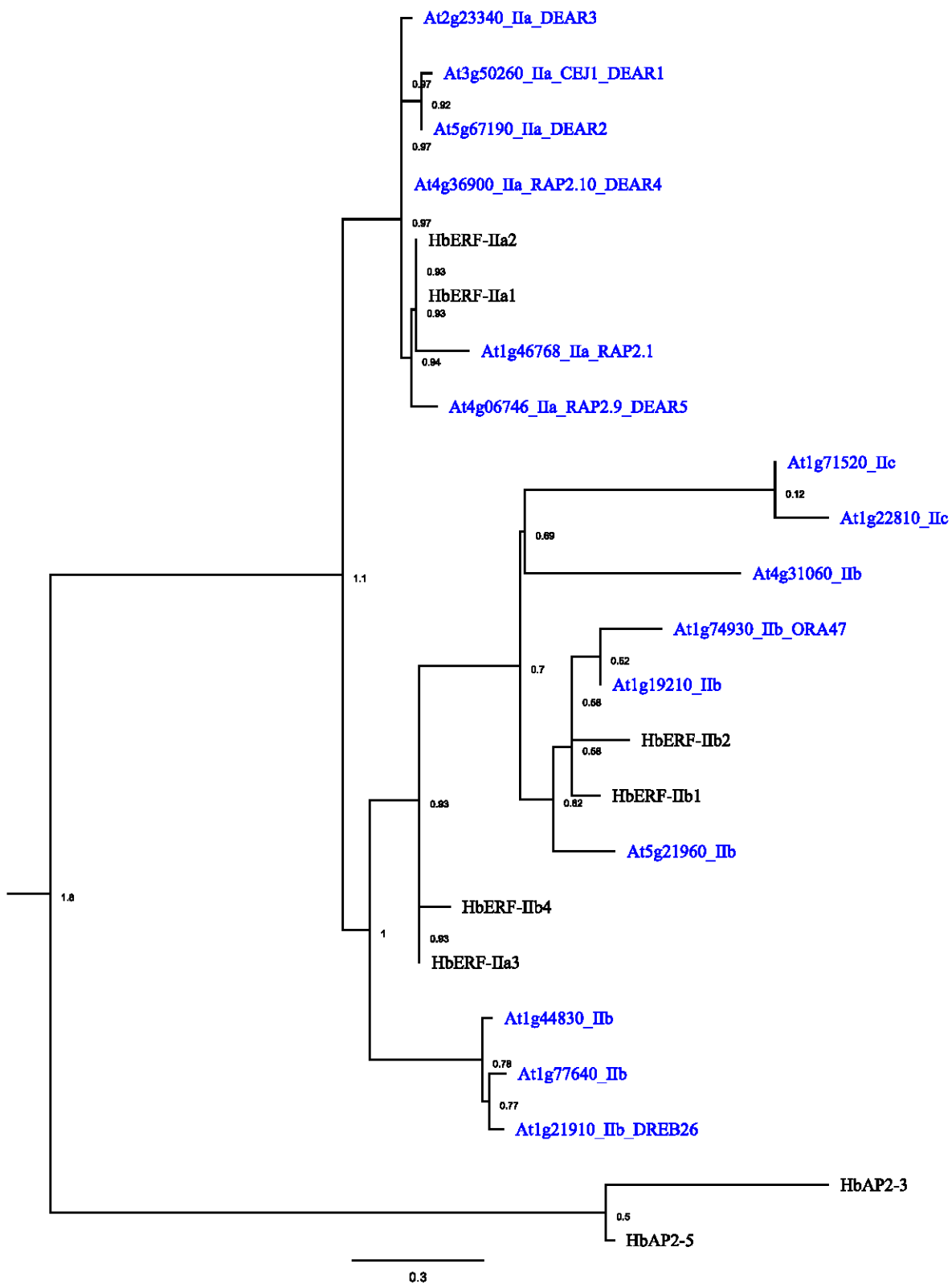

**Figure 2**

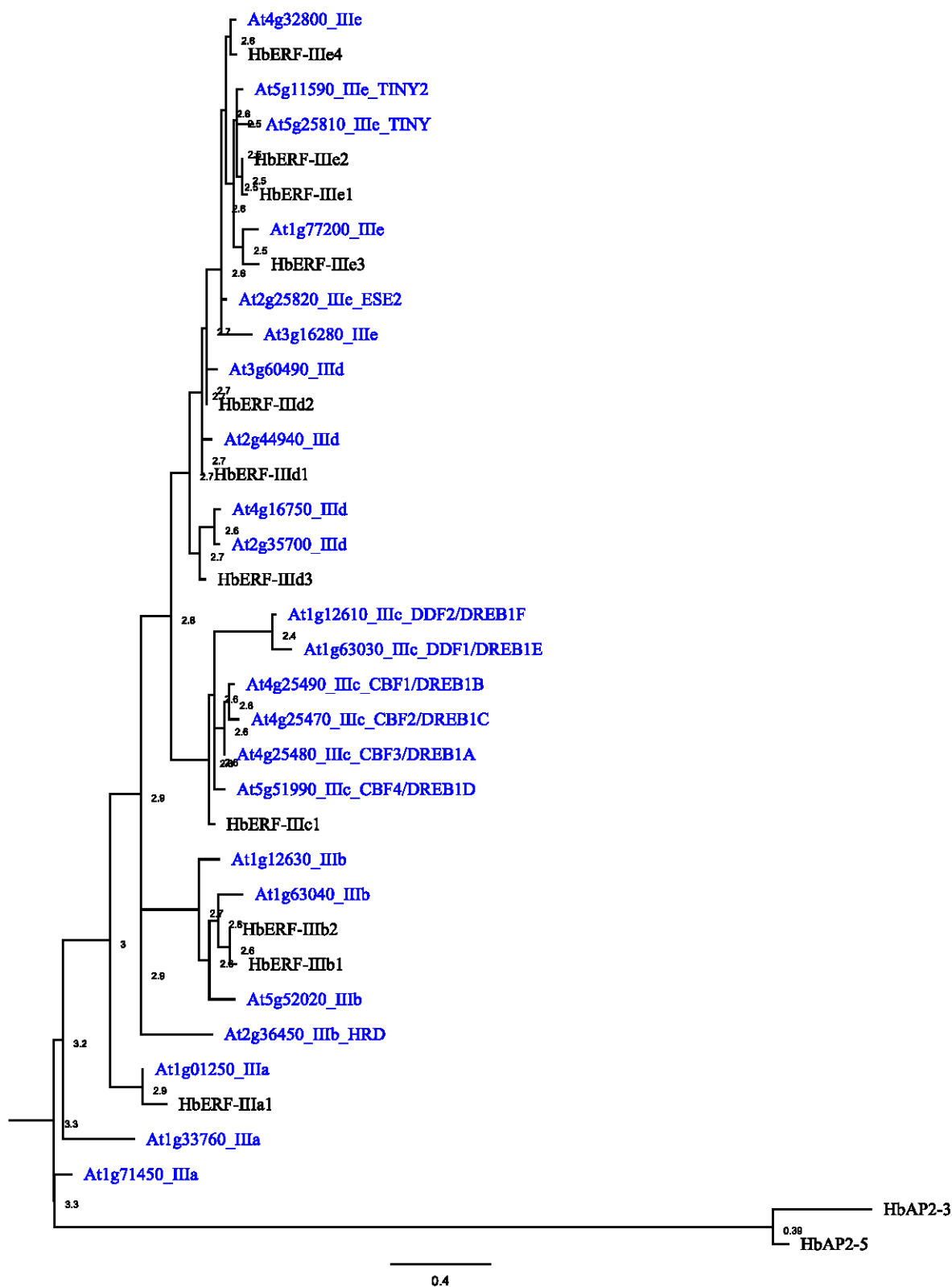

**Figure 3**

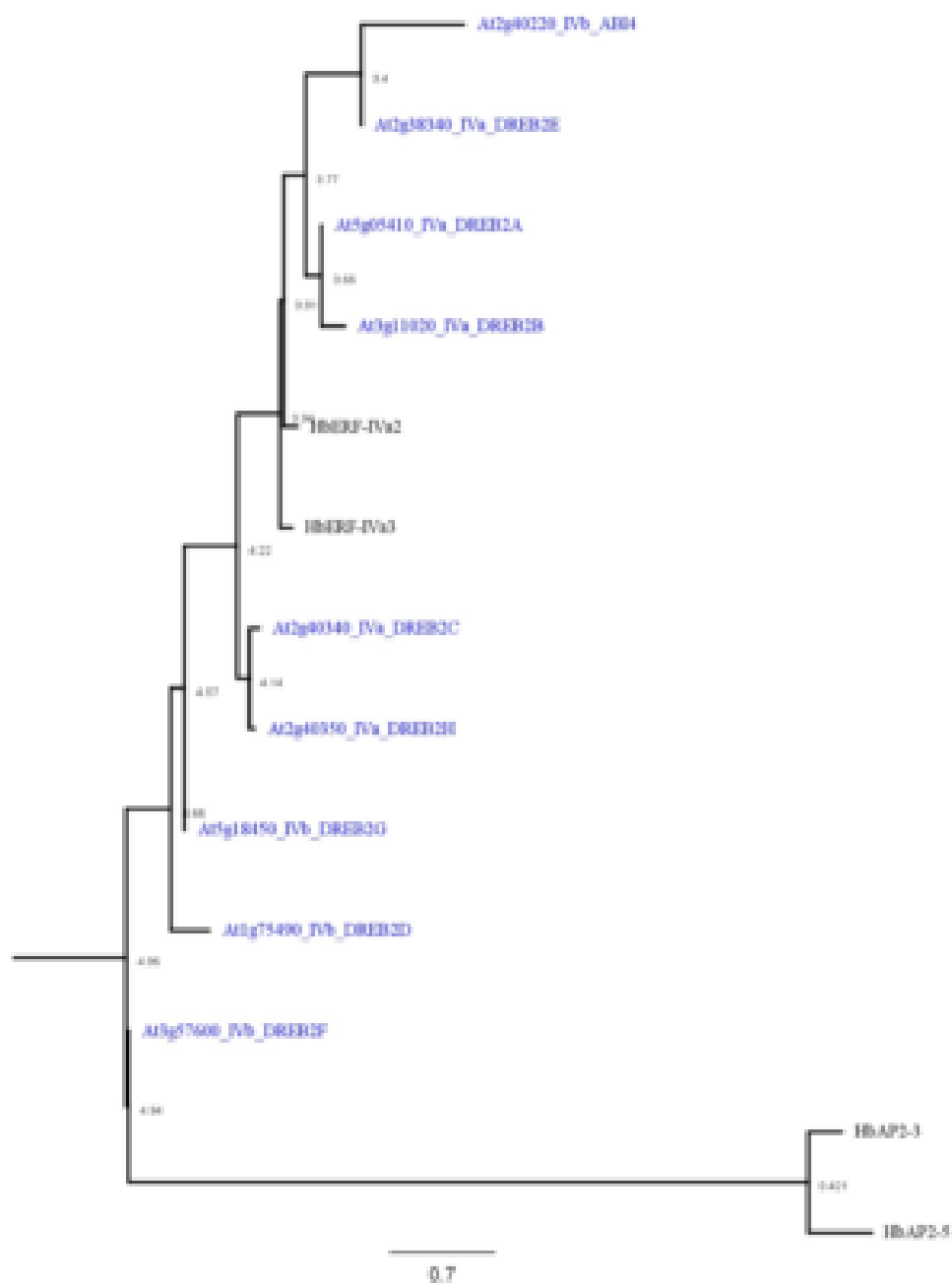

**Figure 4**

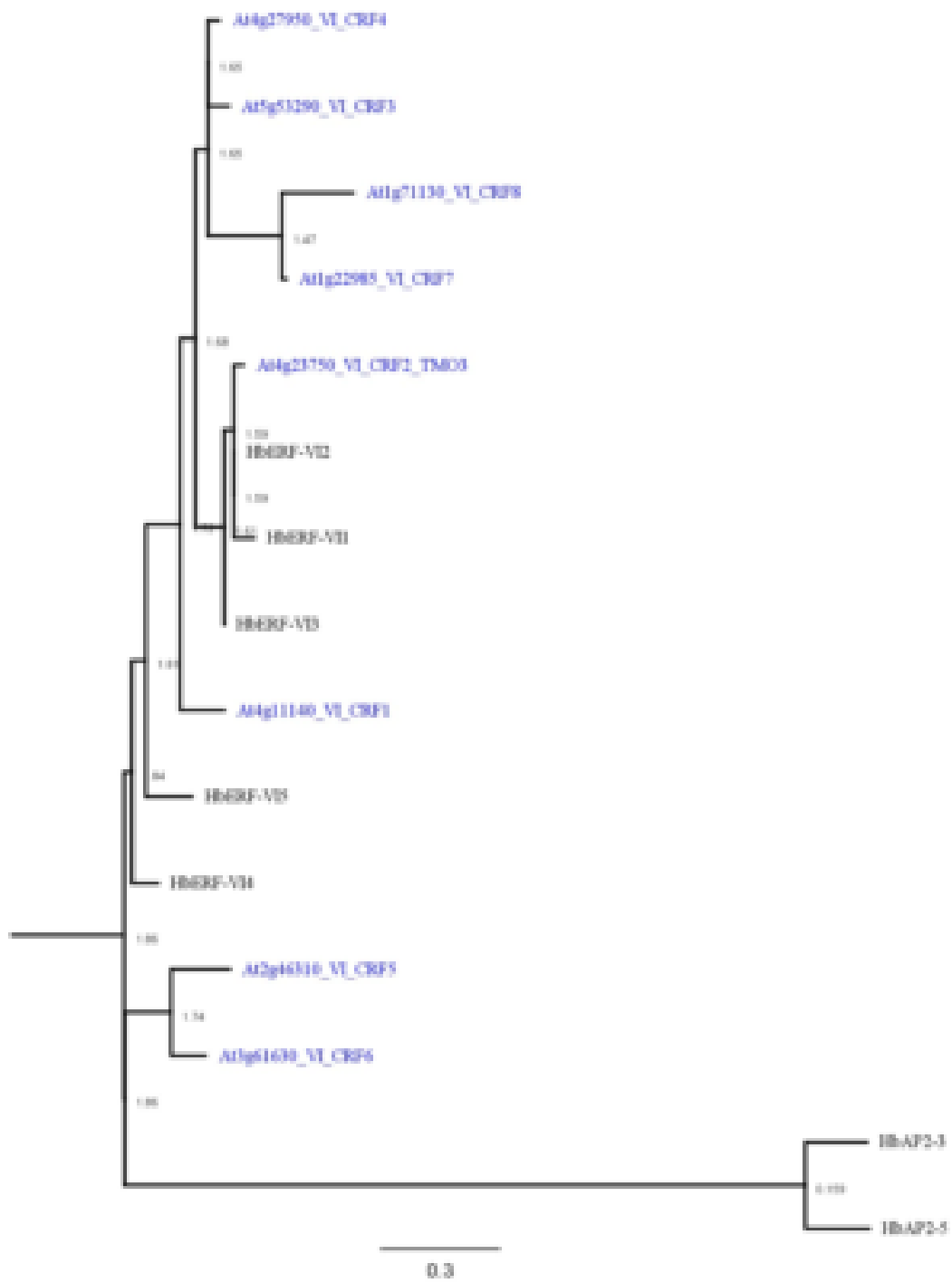

**Figure 5**

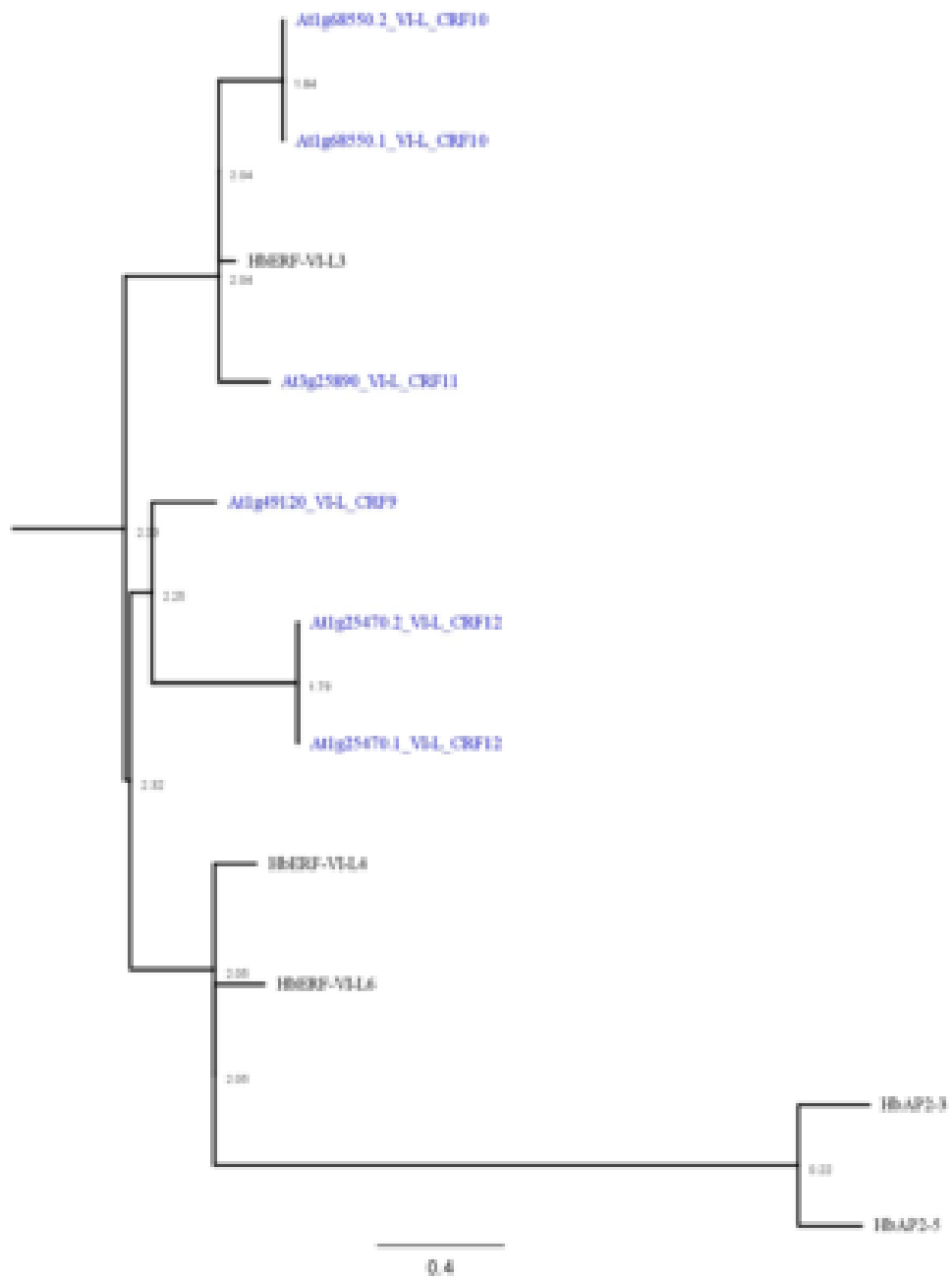

**Figure 6**

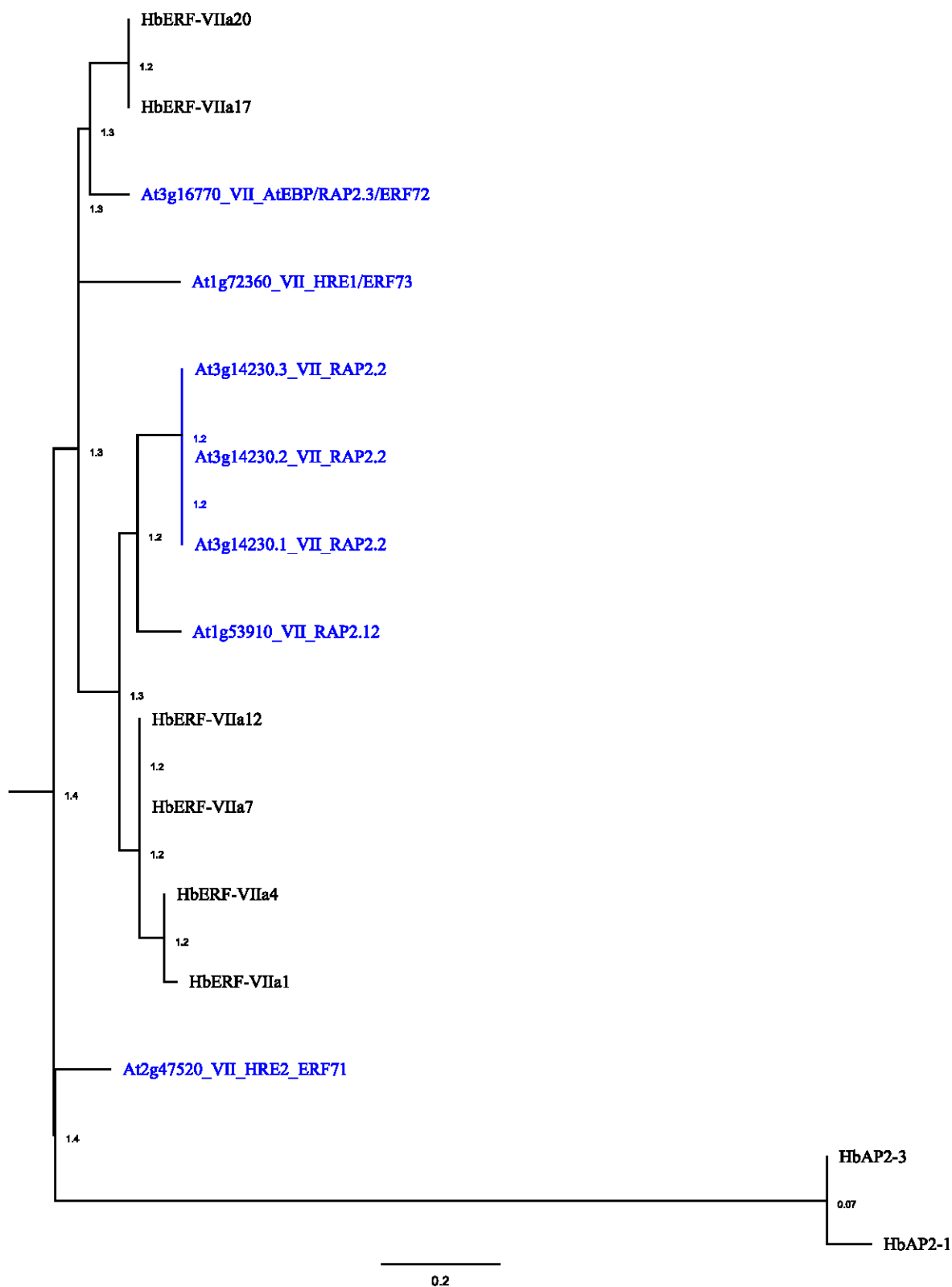

**Figure 7**



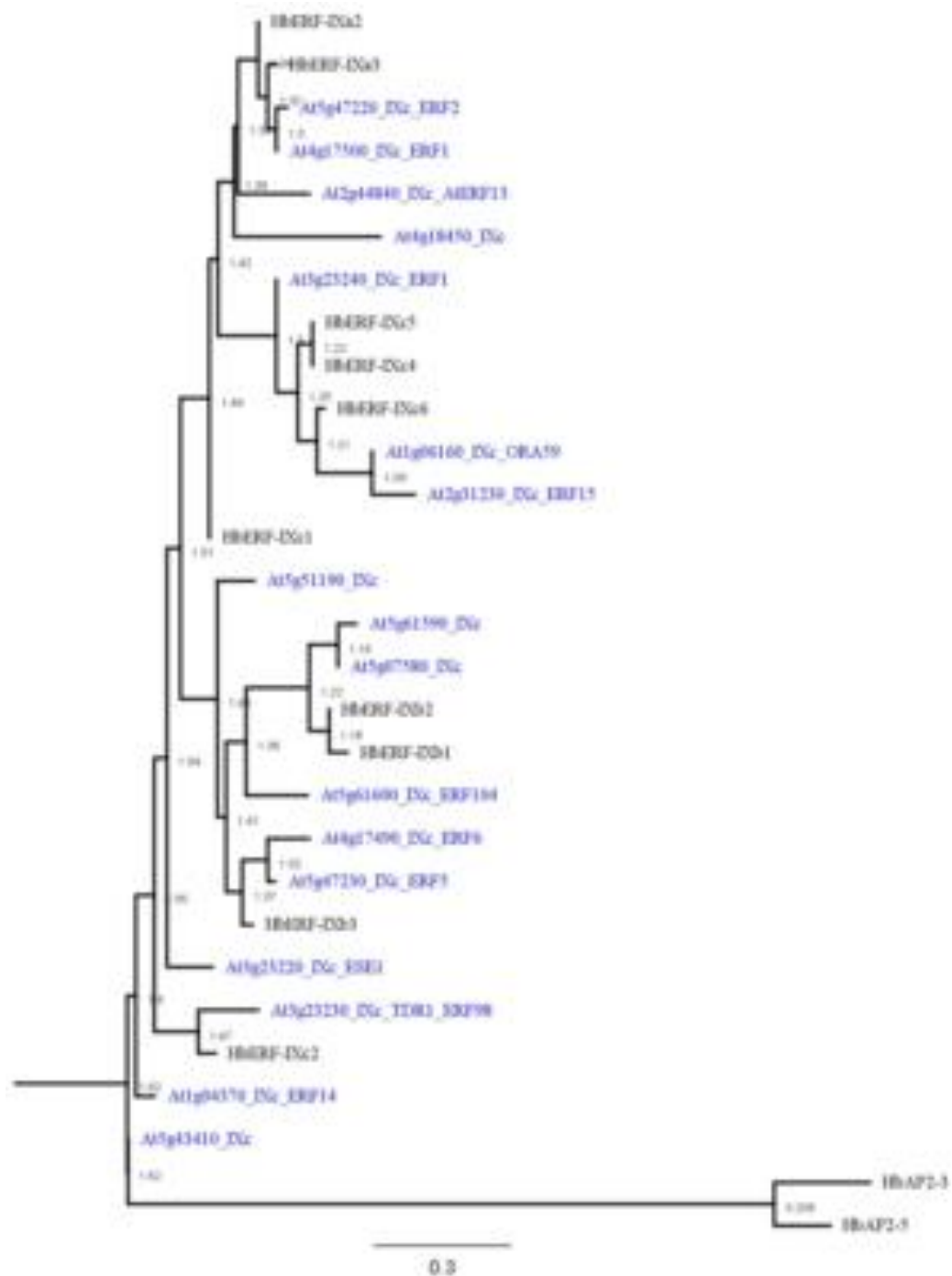

**Figure 9**

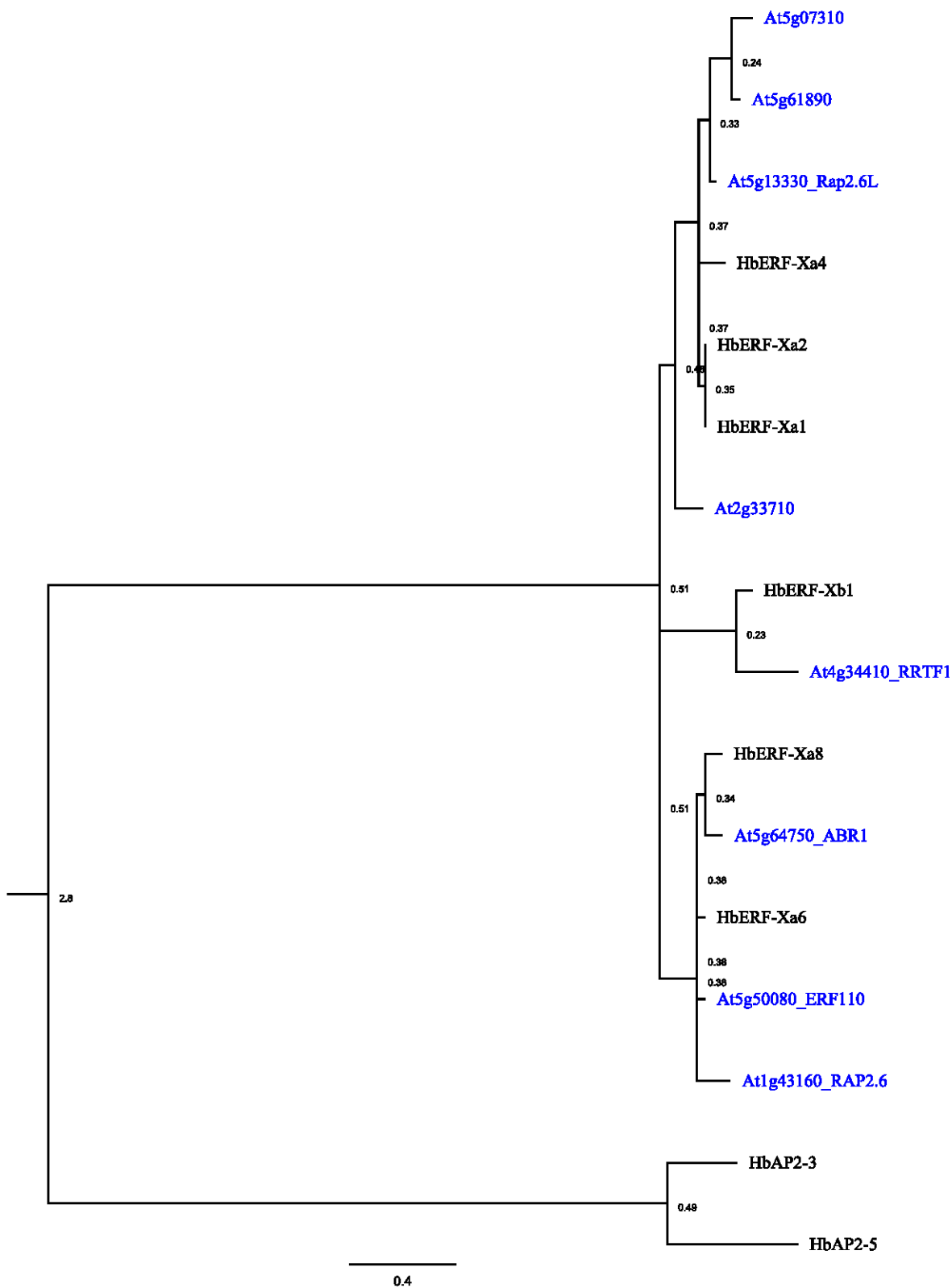

Figure 10

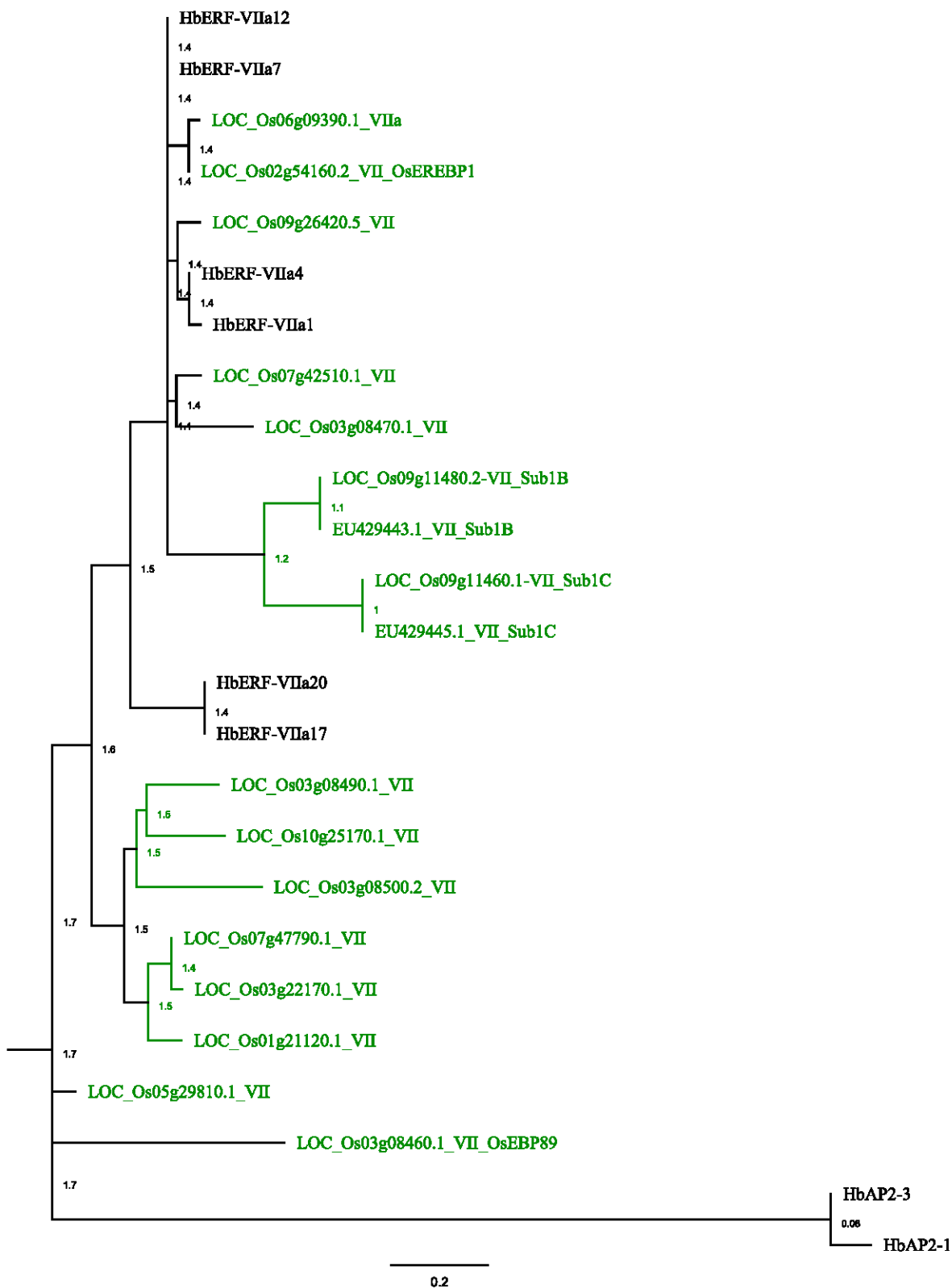

**Figure 11**

Supplement: File S1 — Contains Figure S1, Phylogenetic tree of ERF group I. The deduced amino acid sequences of the AP2 domain from Hevea (black letter) and Arabidopsis (blue letter) were aligned using Muscle, and the phylogenetic tree was constructed using PhyML with an LG+T model. Expression marker genes are indicated in bold letters. Figure S2, Phylogenetic tree of ERF group II. The deduced amino acid sequences of the AP2 domain from Hevea (black letter) and Arabidopsis (blue letter) were aligned using Muscle, and the phylogenetic tree was constructed using PhyML with an LG+T model. Expression marker genes are indicated in bold letters. Figure S3, Phylogenetic tree of ERF group III. The deduced amino acid sequences of the AP2 domain from Hevea (black letter) and Arabidopsis (blue letter) were aligned using Muscle, and the phylogenetic tree was constructed using PhyML with an LG+T model. Expression marker genes are indicated in bold letters. Figure S4, Phylogenetic tree of ERF group IV. The deduced amino acid sequences of the AP2 domain from Hevea (black letter) and Arabidopsis (blue letter) were aligned using Muscle, and the phylogenetic tree was constructed using PhyML with an LG+T model. Expression marker genes are indicated in bold letters. Figure S5, Phylogenetic tree of ERF group VI. The deduced amino acid sequences of the AP2 domain from Hevea (black letter) and Arabidopsis (blue letter) were aligned using Muscle, and the phylogenetic tree was constructed using PhyML with an LG+T model. Expression marker genes are indicated in bold letters. Figure S6, Phylogenetic tree of ERF group VI-L. The deduced amino acid sequences of the AP2 domain from Hevea (black letter) and Arabidopsis (blue letter) were aligned using Muscle, and the phylogenetic tree was constructed using PhyML with an LG+T model. Expression marker genes are indicated in bold letters. Figure S7, Phylogenetic tree of ERF group VII. The deduced amino acid sequences of the AP2 domain from Hevea (black letter) and Arabido [file pone.0099367.s001.pdf]
